# Classification of Schizophrenia by Functional Connectivity Strength Using Functional Near Infrared Spectroscopy

**DOI:** 10.3389/fninf.2020.00040

**Published:** 2020-10-07

**Authors:** Jiayi Yang, Xiaoyu Ji, Wenxiang Quan, Yunshan Liu, Bowen Wei, Tongning Wu

**Affiliations:** ^1^China Academy of Information and Communications Technology, Beijing, China; ^2^Institute of Electrical Engineering, Chinese Academy of Sciences, Beijing, China; ^3^Peking University Sixth Hospital, Peking University Institute of Mental Health, NHC Key Laboratory of Mental Health (Peking University), National Clinical Research Center for Mental Disorders (Peking University Sixth Hospital), Beijing, China; ^4^School of Computer Science and Technology, Donghua University, Shanghai, China; ^5^School of Computer Science and Technology, Xidian University, Xian, China

**Keywords:** functional near infrared spectroscopy (fNIRS), schizophrenia, functional connectivity strength (FCS), support machine vector, classification

## Abstract

Functional near-infrared spectroscopy (fNIRS) has been widely employed in the objective diagnosis of patients with schizophrenia during a verbal fluency task (VFT). Most of the available methods depended on the time-domain features extracted from the data of single or multiple channels. The present study proposed an alternative method based on the functional connectivity strength (FCS) derived from an individual channel. The data measured 100 patients with schizophrenia and 100 healthy controls, who were used to train the classifiers and to evaluate their performance. Different classifiers were evaluated, and support machine vector achieved the best performance. In order to reduce the dimensional complexity of the feature domain, principal component analysis (PCA) was applied. The classification results by using an individual channel, a combination of several channels, and 52 ensemble channels with and without the dimensional reduced technique were compared. It provided a new approach to identify schizophrenia, improving the objective diagnosis of this mental disorder. FCS from three channels on the medial prefrontal and left ventrolateral prefrontal cortices rendered accuracy as high as 84.67%, sensitivity at 92.00%, and specificity at 70%. The neurophysiological significance of the change at these regions was consistence with the major syndromes of schizophrenia.

## Introduction

Schizophrenia is a kind of psychiatric disorder characterized by a series of positive/psychotic (e.g., hallucinations and delusions), negative/deficit (e.g., insufficiency of thought and loss of motivation), and cognitive (e.g., impairment of memory and attention) symptoms (Buckley et al., 2009). Conventionally, clinical diagnostic criteria are predominately based on the relative subjective approaches, for example, according to the diagnostic manuals (American Psychiatric Association, 1994). With the development of neuroimaging, a number of objective methods to identify schizophrenia patients have emerged, e.g., single photon emission computed tomography (SPECT; Gordon et al., 1994), diffusion tensor imaging (DTI; Ohtani et al., 2014), functional magnetic resonance imaging (fMRI; Weiss et al., 2004; Deng et al., 2017; Tréhout et al., 2017), and functional near infrared spectroscopy (fNIRS; Kubota et al., 2005; Rosenbaum et al., 2017).

fNIRS is a noninvasive hemodynamic imaging technique used to assess functional activities in the human brain (Hoshi, 2003). It detects the concentration of oxygenated hemoglobin (Oxy-Hb) and deoxygenated hemoglobin (Deoxy-Hb) by measuring the absorption and reflection of specific near infrared spectrums in the cortices during tasks. Compared to other neuroimaging instruments, fNIRS has the benefit of being low cost with a high portability. These advantages have enabled its application in the diagnosis of schizophrenia, which was mainly based on the effect of hypofrontality (reduced activation around the bilateral prefrontal cortex) during various verbal fluency tasks (VFTs; Suto et al., 2004; Ehlis et al., 2007; Takizawa et al., 2008; Ji et al., 2020). In practice, the majority of these studies extracted the time-domain features from single or multiple channels of healthy subjects and patients with schizophrenia (Suto et al., 2004; Kanahara et al., 2013; Sugimura et al., 2014; Tian et al., 2019). As a consequence, diverse machine learning classifiers (Li et al., 2015) were trained and distinguished the patients with schizophrenia from the healthy subjects.

In contrast, substantial neuroimaging studies of other modalities have found abnormal dysconnectivity between the prefrontal cortex and temporal cortex in schizophrenia patients (Friston and Frith, 1995; Maguire et al., 2000; Greicius, 2008; Bullmore and Sporns, 2009; Whitfield-Gabrieli et al., 2009), and these experiments using EEG and fMRI have proposed classification methods based on brain network properties (Demirci et al., 2008; Yang et al., 2010; Arbabshirani et al., 2013). Nevertheless, the method on whole-brain network properties cannot be directly applied to fNIRS analysis because the conventional clinical fNIRS only measures signals from the frontotemporal cortex. One fNIRS study discriminated patients with schizophrenia using four global network properties (Song et al., 2016). The achieved overall accuracy was 85.5%, but the local changes could not be investigated with the approach. Hence, the analysis on regional functional connectivity (FC), integrating both the spatial and temporal relation of brain activities, is hypothesized to provide new insights on classifying schizophrenia.

In this article, we provided an FC-based method to identify schizophrenia patients. Oxy-Hb data from 100 schizophrenia patients and 100 healthy subjects during VFT were used in the experiment. functional connectivity strength (FCS) from single channel, from the ensemble 52 channels, from the dimensional reduced 52 channels, and from different combinations of 2–5 channels were used to trained four popular classifiers (Linear Discriminant Analysis: LDA, k-Nearest Neighbor: KNN, Gaussian Processes classifier: GPC, and Support Vector Machine: SVM), respectively. The best accuracy was 85.00% (LOOCV), with sensitivity as 87.00% (LOOCV) and specificity as 83.00% (LOOCV), by using FCS from three channels. Theneurophysiological significance was discussed. The FCS-based method provided a new and effective approach for schizophrenic identification.

## Materials and Methods

### Subject

The Oxy-Hb dataset included 100 schizophrenic (male/female: 50/50, 33.81 ± 11.52 years old and ranging from 18 to 53 years old) and 100 healthy subjects (male/female: 47/53, 34.43 ± 12.36 years old and ranging by 18–78 years old) who were recruited from Peking University Sixth Hospital. The diagnosis for schizophrenia was based on DSM-IV and conducted by two clinical doctors. All subjects were native Chinese speakers and right-handed. This study was carried out in conformity with the Declaration of Helsinki and was sustained by the ethics committee of Peking University Sixth Hospital. All subjects provided written consent after being fully informed of the procedures in the study.

### VFT Experiment

**Figure 1:**
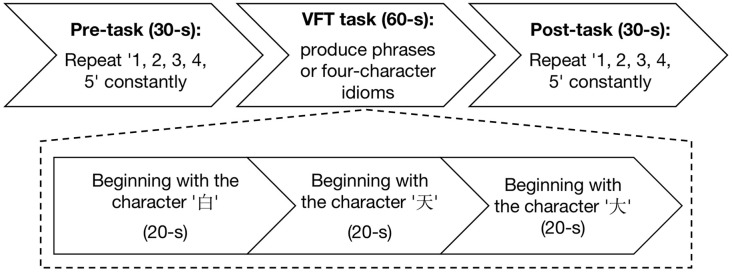
Experimental flowchart. The experiment has three procedures: 30-s pre-task, 60-s verbal fluency task (VFT) task, and 30-s post-task.

The experiment was conducted in a quiet room and no entry was permitted during the experiment. The Chinese VFT (Quan et al., 2015) was initiated by a 30-s pre-task baseline period, followed by a 60-s task period and a 30-s post-task baseline period (Figure 1). There was a screen 1 m in front of the participants. During the pre-task and post-task baseline periods, the participants were asked to stare at the center of the screen and count from 1 to 5. During the 60-s task period, three Chinese characters (“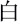,” “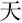,” and “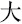,” indicating white, sky, and big, respectively) were displayed on the screen and changed every 20 s. The participants were instructed to produce as many phrases or four-character idioms starting with these characters as they could.

### fNIRS Measurement and Data Preprocessing

**Figure 2:**
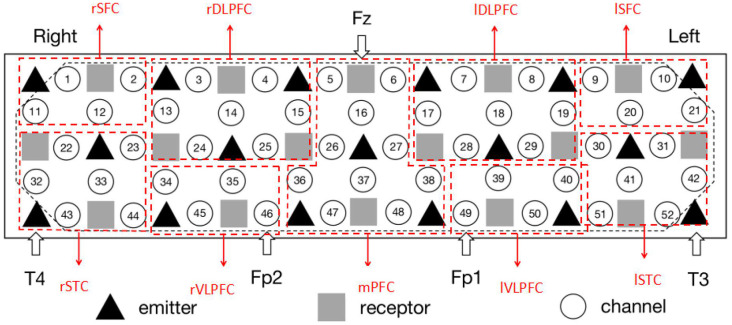
The setting of functional near infrared spectroscopy (fNIRS) probe and channel. Hexagons stand for near-infrared light emitters, diamonds stand for near-infrared light receptors, and cycles stand for fNIRS channels. T3, T4, Fp1, Fp2, and Fz are the electrode positions in the international 10-20 system. rSFC, right superior frontal cortex; rSTC, right superior temporal cortex; rDLPFC, right dorsolateral prefrontal cortex; rVLPFC, right ventrolateral prefrontal cortex; mPFC, medial prefrontal cortex; lDLPFC, left dorsolateral prefrontal cortex; lVLPFC, left ventrolateral prefrontal cortex; lSFC, left superior frontal cortex; lSTC, left superior temporal cortex. The placement is in line with the configuration of Schecklmann et al. (2010).

The measurement was conducted using a 52-channel near infrared spectrometer (ETG-4000, Hitachi Medical Co., Japan). The instrument had 33 probes (17 emitters and 16 detectors; Figure 2). The positioning of the receivers emitters was referred to an international 10-20 system of Electroencephalography (Oostenveld and Praamstra, 2001). To specify, the detector between Channel #5 and #6 was located at Fz, the emitters close to #43 and #52 were fitted around T4 and T3, and #46 and #49 were placed in Fp2 and Fp1, respectively. The measurement area covered the bilateral prefrontal and temporal cortices (Figure 2). The separation between the channels was 3 cm. In the experiments, each subject was measured with 120 s (30 s pre-task baseline, 60 s VFT and 30 s post-task baseline) at a sampling rate of 10 Hz. Hence, there were 1,200 signal points for each channel per subject. The measured Oxy-Hb signal was organized as a matrix with 300 × 1,200 × 52 (number of subjects × signal points × amount of channels).

The raw Oxy-Hb data were preprocessed through a band-pass filter of 0.009–0.08 Hz to remove the motion artifacts. The least square method was used to eliminate and remove the linear trend from the Oxy-Hb signals. MATLAB toolkit HomER2 (Huppert et al., 2009) was used to preprocess the original data.

### Feature Extraction for Classification

The conventional classification methods usually utilized the time-domain features (for example, mean amplitude of Oxy-Hb during VFT). Frontal functional dysconnectivity is a salient feature of schizophrenia but it has not yet been applied for identifying schizophrenia. FCS was selected to characterize the effect and the following steps were used to obtain the value:

(1) Pearson’s correlation among the data from 52 channels was calculated by:

(1) $$r_{\text{xy}}=\frac{\sum x_{i}y_{i}-n\bar{x}\bar{y}}{(n-1)S_{\text{x}}S_{\text{Y}}}$$

where $\bar{x}$, $\bar{y}$ are the mean, and *S*_x_, *S*_y_ are the standard deviations of the measured data *x*_i_ and *y*_i_, respectively; *n* is the number of the data.

(2) FCS was calculated by:

(2) $$FCS=\frac{\sum_{y}r_{x,y}}{51}$$

As a consequence, 52 FCSs were derived *per* subject. We assessed the results from three kinds of approaches to identify schizophrenia.

-   Single-feature: FCS from single channel
-   Ensemble 52 FCSs: FCSs from 52 channels with or without dimensional reduction techniques
-   Combine-features: FCS from the combination of several single-features

Four widely used classifiers in schizophrenic identification, LDA, GPC, KNN, and SVM, were trained and applied in the study. Since the details of these classifiers were extensively discussed previously (Mourão-Miranda et al., 2005; Yoon et al., 2007; Azechi et al., 2010; Tanaka and Katura, 2011; Dai et al., 2012; Arbabshirani et al., 2013; Hahn et al., 2013), we will not repeat them again. The MATLAB toolkits, GPML (v3.4^1^; Rasmussen and Nickisch, 2010), LIBSVM (v3.1.2^2^, and LDA (V1.0.0.0^3^) were used in the analysis. KNN was realized by MATLAB function KNNCLASSIFY.

Note that the major parameters of these toolkits and functions used default or empirical values, with the exceptions of:

-   KNN: *k* = (100)^1/2^ = 10; Euclidean distance is adopted to calculate the distances between the unlabeled sample and the labeled training samples. Traditionally, the Euclidean distance is appropriate when the issue included mutually correlated observations. As such, this distance needs to consider every variable and does not remove redundancies. The situation is very similar to our situation: the data from 52 channels are highly correlated and none can be simply removed. k is to set $k=\sqrt{n}$ The method has been proposed by Mitra et al. (2002);
-   SVM: RBF kennel; C and gamma were optimized by automated grid search and evaluated *via* 10-fold cross-validation. The optimization was conducted per case and the best RBF factors were provided along with the results.

### Evaluation of the Classification Performance

To evaluate the performance of the individual classifier, both leave-one-out cross-validation (LOOCV) and 10-fold/ 20-fold CV were used to estimate the performance of the classifier. The subjects involved in the experiments were schizophrenia patients (positive, P) and the healthy controls (negative, N). The true positive (TP) and the true negative (TN) are the number of patients and healthy people being correctly classified, respectively. The false positive (FP) is the number of healthy people being classified as patients. The false negative (FN) is the number of patients being classified as healthy people. The performance of the classification method was assessed in terms of accuracy, sensitivity, and specificity as shown in:

(3) $$accuracy=\frac{TP+TN}{\left(TP+FP+FN+TN\right)}$$

(4) $$sensitivity=\frac{TP}{\left(TP+FN\right)}$$

(5) $$specificity=\frac{TN}{\left(FP+TN\right)}$$

## Results

### FC Matrices

**Figure 3:**
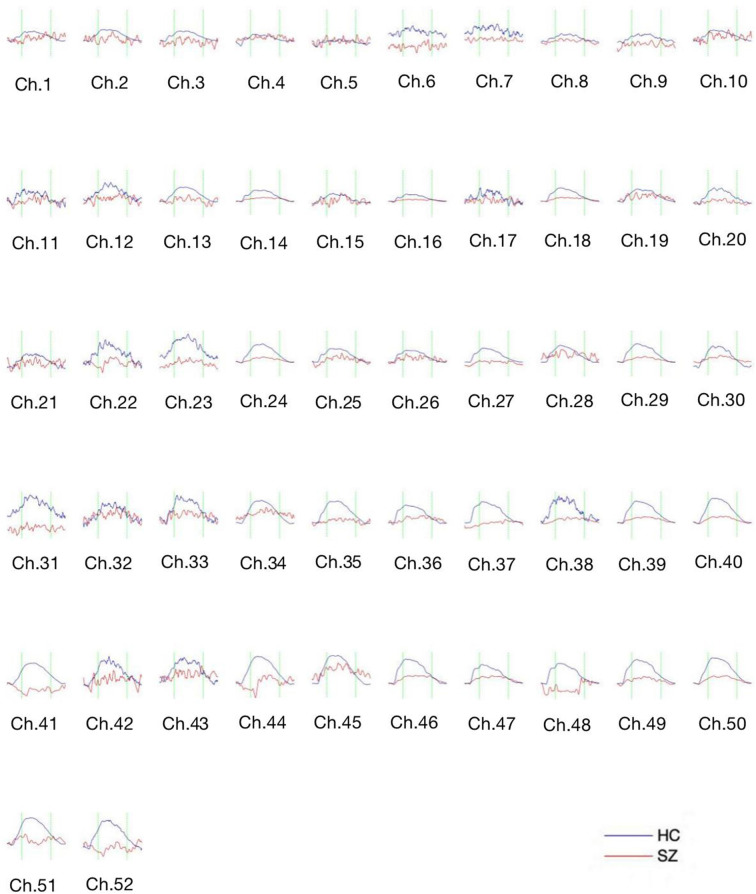
The concentration curve of Oxy-Hb from 52 channels averaged from the healthy controls (blue lines) and the schizophrenic group (red lines). SZ, schizophrenic patients; HC, healthy controls.

**Figure 4:**
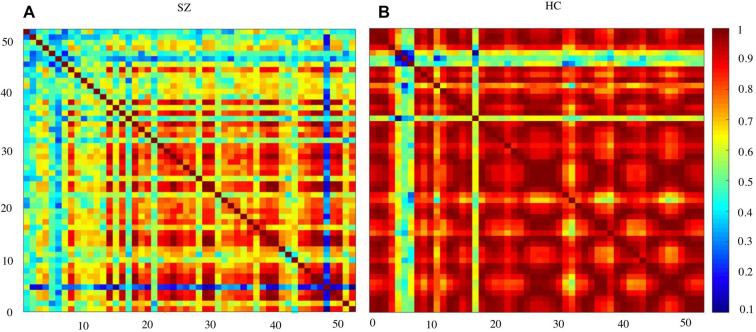
Functional connectivity (FC) matrices averaged over schizophrenia group and healthy controls. SZ, schizophrenic patients **(A)**; HC, healthy controls **(B)**.

Figure 3 shows the waveform of Oxy-Hb from 52 channels. The results were averaged across the healthy control and schizophrenic group. The reduced Oxy-Hb during VFT was obvious in patients with schizophrenia, being consistent with previous literature. The derived FCs were mapped in Figure 4. It was revealed that functional connections with high intensity were observed in the healthy controls, indicating dysconnectivity of schizophrenia.

### Classification Results

#### Single-FCS Results

**Figure 5:**
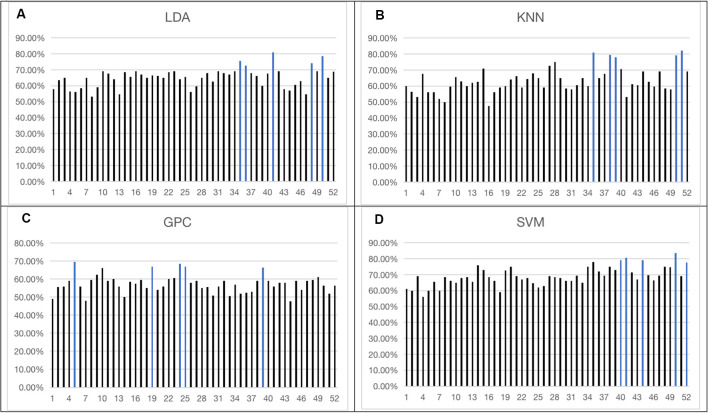
Overall accuracy achieved by single-channel feature using four classifiers [LDA **(A)**, KNN **(B)**, GPC **(C)**, SVM **(D)**]. The top five channels were indicated by blue (the optimized C and gamma of the top five channels of SVM are shown in the Supplementary Figures 1–5). LDA, Linear Discriminant Analysis; KNN, k-Nearest Neighbor; GPC, Gaussian Processes classifier.

The overall accuracy from the top five channels to identify schizophrenia is shown in Figure 5 and is summarized in Table 1. LDA, KNN, and SVM demonstrated similar overall levels of accuracy, i.e., LDA: 72.50–81.00% (LOOCV), KNN: 78.00–82.00% (LOOCV), and SVM: 77.50–83.50% (LOOCV). GPC had the lowest accuracy at 67.00–69.50% (LOOCV). In terms of spatial distribution of the channels, although LDA, KNN, and SVM demonstrated laterality (left or right sidedness), in general, the best channels identified by these three classifiers were on the ventral part of the frontal cortices. In contrast, GPC utilized the FCS from the dorsal channels.

**Table 1:** Top five channels with functional connectivity strength (FCS) representing the best overall accuracy from the four classifiers (the optimized C and gamma of five channels of Support Vector Machine (SVM) are shown in Supplementary Figures 1–5).

LDA	Channel	Brain region	Accuracy
	#41	lSTC	81.00%
	#50	lVLPFC	78.50%
	#35	rVLPFC	75.50%
	#48	mPFC	74.00%
	#36	mPFC	72.50%
**KNN**			
	#51	lSTC	82.00%
	#35	rVLPFC	81.00%
	#38	mPFC	79.50%
	#50	lVLPFC	79.00%
	#39	lVLPFC	78.00%
**GPC**			
	#5	mPFC	69.50%
	#24	rDLPFC	68.50%
	#39	lVLPFC	66.50%
	#25	rDLPFC	67.00%
	#19	lDLPFC	67.00%
**SVM**			
	#50	lVLPFC	83.50%
	#41	lSTC	80.50%
	#40	lVLPFC	79.00%
	#44	rSTC	79.00%
	#52	lSTC	77.50%

#### 52-FCS Results

**Figure 6:**
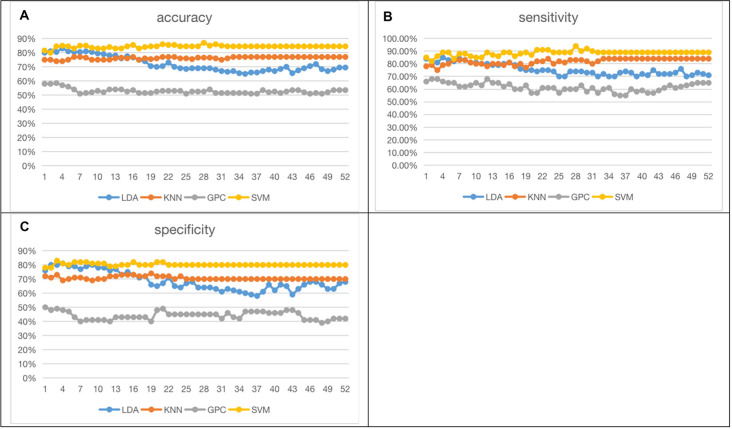
Fifty-two functional connectivity strength (FCS) results [accuracy **(A)**, sensitivity **(B)**, specificity **(C)**; the optimized C and gamma of best accuracy as 86.50% of SVM are shown in Supplementary Figure 6].

Figure 6 shows the 52-feature results from different classifiers. Again, GPC achieved the lowest results (accuracy at 51.00% (LOOCV), with sensitivity at 55.00% (LOOCV) and specificity at 47.0% (LOOCV)). The other three classifiers had similar performances whilst SVM slightly outperformed the other two. In summary, SVM achieved the best accuracy at 86.50% (LOOCV), sensitivity at 91.00% (LOOCV), and specificity at 82% (LOOCV). LDA had the best accuracy at 83.00% (LOOCV), sensitivity at 85.00% (LOOCV), and specificity at 81.00% (LOOCV). KNN yielded the best accuracy at 77.00% (LOOCV), sensitivity at 84.00% (LOOCV), and specificity at 70.00% (LOOCV). The performance of the classifiers initially increased with the numbers of channels but stabilized when more channels were taken into consideration. It may indicate the existence of redundancy in this feature space.

#### Dimensional Reduced 52-FCS Results

Principal component analysis (PCA) can convert multiple observations of potentially correlated variables into a set of linearly independent components. It implemented singular value decomposition to reduce the dimensionality of a dataset that consisted of a large number of interrelated variables, while retaining as much variation present in the dataset as possible (Abdi and Williams, 2010). For comparison, we also used two other variants of PCA in the analysis: Kernel PCA and Sparse PCA. Kernel PCA uses various kernel functions to project datasets into a higher dimensional feature space, where it is linearly separable. We selected Gaussian kernel in this case. It was realized by MATLAB function KernelPca.m (Kitayama, 2020). Sparse PCA is implemented on the basis of the inverse power method for nonlinear eigenproblems, which is introduced in detail by Hein and Bühler (2010). Moreover, the deflation scheme proposed by Bühler (2015) is adopted to compute multiple principal components. It was realized by the free software sparsePCA developed by Matthias Hein and Thomas Bühler [Copyright 2010–2020 Thomas Bühler and Matthias Hein (hein@cs.uni-saarland.de). Machine Learning Group, Saarland University, Germany^4^].

We derived the first 21 principal components representing cumulative rates exceeding 93.4%. Classification based on the selected principal components is shown in Table 2. The results of 10-fold and 20-fold cross validation were provided for comparison.

**Table 2:** Classification performance of leave-one-out cross-validation (LOOCV), 10-fold, and 20-fold by using three dimensionality reduction methods (the optimized C and gamma of SVM are shown in Supplementary Figures 7–15).

		LOOCV	10-fold	20-fold
LDA	Accuracy (PCA KernelPCA SparsePCA)	80.50% 80.00% 82.00%	79.00% 80.50% 80.50%	81.50% 82.00% 82.50%
	Sensitivity (PCA KernelPCA SparsePCA)	82.00% 81.00% 83.00%	81.00% 82.00% 83.00%	83.00% 84.00% 83.00%
	Specificity (PCA KernelPCA SparsePCA)	79.00% 79.00% 81.00%	77.00% 79.00% 78.00%	80.00% 80.00% 82.00%
KNN	Accuracy (PCA KernelPCA SparsePCA)	79.00% 82.00% 79.50%	80.50% 80.50% 82.00%	80.00% 81.00% 80.50%
	Sensitivity (PCA KernelPCA SparsePCA)	82.00% 83.00% 81.00%	81.00% 82.00% 84.00%	82.00% 83.00% 81.00%
	Specificity (PCA KernelPCA SparsePCA)	76.00% 81.00% 78.00%	80.00% 79.00% 80.00%	78.00% 79.00% 80.00%
GPC	Accuracy (PCA KernelPCA SparsePCA)	72.00% 73.50% 75.00%	68.50% 70.00% 70.50%	70.00% 71.50% 70.50%
	Sensitivity (PCA KernelPCA SparsePCA)	73.00% 75.00% 77.00%	70.00% 72.00% 72.00%	72.00% 73.00% 72.00%
	Specificity (PCA KernelPCA SparsePCA)	71.00% 72.00% 73.00%	67.00% 68.00% 70.00%	68.00% 70.00% 69.00%
SVM	Accuracy (PCA KernelPCA SparsePCA)	87.50% 89.00% 88.00%	85.50% 87.00% 88.50%	87.50% 86.00% 89.50%
	Sensitivity (PCA KernelPCA SparsePCA)	89.00% 90.00% 89.00%	86.00% 88.00% 89.00%	86.00% 87.00% 90.00%
	Specificity (PCA KernelPCA SparsePCA)	86.00% 88.00% 87.00%	85.00% 86.00% 88.00%	89.00% 85.00% 89.00%

#### Combined FCS Results

Further effort was made to assess the capability of schizophrenic identification using a certain combination of the channels. Since SVM yielded the best overall accuracy, the experiments were conducted only using this classifier. FCSs from 2, 3, 4, and 5 channels were selected from the five channels presenting the top capability on schizophrenic identification (presented in Figure 5). The results are shown in Table 3. Classification using FCS from three channels can achieve accuracy at 85.00% (LOOCV), sensitivity at 87.00% (LOOCV), and specificity at 83.00% (LOOCV).

**Table 3:** Overall accuracy of different combinations of FCS using SVM (the optimized C and gamma are shown in Supplementary Figures 16–41).

Selection of the channels					Accuracy	Sensitivity	Specificity
#40	#41	#44	#50	#52			
x	x				82.00%	84.00%	80%
x		x			81.50%	82.00%	81%
x			x		80.50%	82.00%	79%
x				x	83.00%	85.00%	81%
	x	x			78.50%	79.00%	78%
	x		x		82.00%	83.00%	79%
	x			x	83.00%	83.00%	83%
		x	x		79.00%	80.00%	78%
		x		x	80.50%	81.00%	80%
			x	x	83.50%	85.00%	82%
x	x	x			81.50%	83.00%	80%
x	x		x		85.00%	87.00%	83%
x	x			x	84.00%	86.00%	82%
x		x	x		82.00%	82.00%	80%
x			x	x	84.50%	85.00%	84%
x		x		x	83.00%	85.00%	81%
	x	x	x		80.00%	82.00%	78%
	x	x		x	79.50%	81.00%	78%
	x		x	x	80.00%	81.00%	79%
		x	x	x	81.00%	84.00%	78%
x	x	x	x		83.50%	86.00%	81%
x	x	x		x	83.00%	84.00%	82%
x	x		x	x	84.50%	85.00%	84%
x		x	x	x	82.00%	85.00%	79%
	x	x	x	x	83.00%	84.00%	82%
x	x	x	x	x	84.50%	86.00%	83%

## Discussions

Schizophrenia has been considered a disorder of connectivity between various brain units (Elvevåg and Goldberg, 2000). The connections were found to be reduced by schizophrenia, as shown in Figure 4. This finding was consistent with studies using other imaging modalities (Bellani et al., 2010; Deng et al., 2017).

FCS measures the connectivity across different brain units, so as to identify the hubs playing important roles in information processing and communication during cognitive tasks (van den Heuvel and Sporns, 2013; Mears and Pollard, 2016). As shown in Table 1, the capability of discriminating schizophrenia was evident for the FCS at VLPFC and mPFC. mPFC relates to decision making and short- and long-term memory (Euston et al., 2012), and coordinates VLPFC and DLPFC functions (Peng et al., 2018). The neurophysiological functions of this cortex are associated with the symptoms of schizophrenia. The left VLPFC associates with the production of articulate language and in nonlinguistic tasks (Hickok and Poeppel, 2004, 2007), while the right VLPFC plays a role in linking working memory with episodic memory and in a series of complicated social behaviors (He et al., 2020). The reduced FCS of VLPFC in patients with schizophrenia may relate to the impairment of both verbal skills and social functions, which are the major symptoms of schizophrenia. In contrast, some channels were at STC, which mediates spatial awareness and exploration (Karnath, 2001). To summarize, these changes during VFT corresponded to the perturbed performance of schizophrenia patients (difficulty or incapability to produce four-character idioms).

PCA reduced the dimension of the feature space and saved the computational cost, while achieving satisfactory accuracy. The disadvantage of PCA was that the principle components could not be attributed to the data from the specific channel, thus concealing the regional neurophysiological changes. Using the FCS from three channels, the achieved performance was comparable to the current results: accuracy at 70–86%, sensitivity at 70–84%, and specificity at 65–93% (Arbabshirani et al., 2013; Chuang et al., 2014; Li et al., 2015; Pina-Camacho et al., 2015; Song et al., 2017). The method was not calculated from the time-domain values on single or multiple channels. It means that reliable results could be provided when integrated with the time-domain approaches.

There are some limitations to the present study. First, the individual schizophrenic episode was not identified and taken into analysis. However, it may have implications on the effected sites (Zhu et al., 2010). Second, the patients receiving medications and physical treatment were not ruled out from the study. Although previous studies have revealed a negligible medication effect on fNIRS signals, investigations of drug-free patients or of those receiving physical treatment (e.g., transcranial magnetic stimulation, electroconvulsive therapy, and neurofeedback) will be needed to allow further clinical applications of fNIRS (Fujita et al., 2011; Mihara et al., 2012; Monden et al., 2012). Third, educational background may have an impact on language ability. In our study, we did not categorize the subjects into more educational background groups because the number of subjects in each group would be sparse. But the two groups matched their educational backgrounds (the schizophrenia group included 14 graduate degrees, 20 undergraduate degrees, 20 college degrees, 31 senior high school degrees, and 15 junior high school degrees, and the healthy group included 15 graduate degrees, 20 undergraduate degrees, 20 college degrees, 30 high school degrees, and 15 junior high school degrees). Lastly, only three machine-learning classifiers, LDA, KNN, and SVM, were used in the study because they were the most popular machine-learning classifiers in discriminating patients with schizophrenia. The comparison of their performance was a topic being widely discussed while the individual performance seemed to be signal- and feature-dependent (Mourão-Miranda et al., 2005; Hahn et al., 2013; Li et al., 2015). In the present study, we conducted a similar comparison. Other classifiers, such as artificial neural networking, has not yet been applied but theoretically could be utilized in the identification of schizophrenia (Zheng et al., 2019a,b, 2020). We will try it in our future study.

## Conclusion

The study proposed an FCS-based method to identify patients with schizophrenia. 52-channel Oxy-Hb data of fronto-temporal fNIRS were obtained during VFT from healthy and schizophrenic subjects. The FCS of each channel was calculated as features for classification. We investigated the performance of different classifiers, from FCS of all the 52 channels or from several channels. The method was in sharp contrast to most previous studies using the time-average data obtained from multiple channels. The classification results were comparable to the existing results. In addition, the method can detect the changes of hubs during VFT, which was in consistency with the symptoms of schizophrenia.

## Data Availability Statement

The datasets presented in this article are not readily available because dataset are strictly restricted to non-commercial uses. Requests to access the datasets should be directed to wutongning@caict.ac.cn.

## Ethics Statement

The studies involving human participants were reviewed and approved by the ethics committee of Peking University Sixth Hospital. The patients/participants provided their written informed consent to participate in this study.

## Author Contributions

TW conceptualized the experiments and supervised their implementation. JY and XJ contributed code to the project. WQ contributed to data acquisition. YL and BW validated the results and visualized them. XJ wrote the first version of the manuscript with input from JY and TN. TW and JY wrote the final version of the manuscript.

## Conflict of Interest

The authors declare that the research was conducted in the absence of any commercial or financial relationships that could be construed as a potential conflict of interest.

## Footnotes

^1^http://www.gaussianprocess.org/gpml

^2^http://www.csie.ntu.edu.tw/~cjlin/libsvm/

^3^https://ww2.mathworks.cn/matlabcentral/fileexchange/29673-lda-linear-discriminant-analysis

^4^http://www.ml.uni-saarland.de
