# Supplementary material for: Classification of Schizophrenia by Functional Connectivity Strength Using Functional Near Infrared Spectroscopy
Source: Front Neuroinform. 2020 Oct 7;14:40. doi: 10.3389/fninf.2020.00040 (PMC7575761; doi:10.3389/fninf.2020.00040)
Supplement: Supplementary file 1 [file Data_Sheet_1.docx]

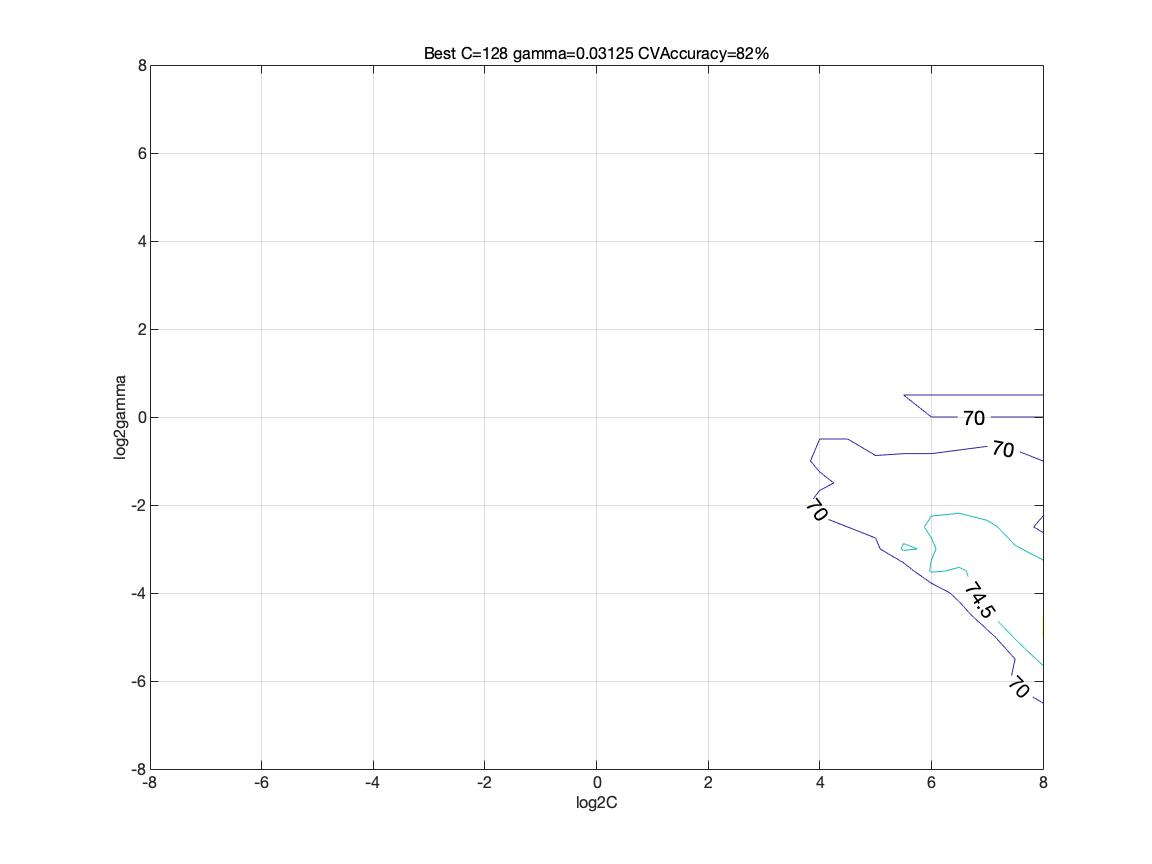


**Figure 1.** CH: 50, the grid searching results of the two RBF parameters used in SVM.


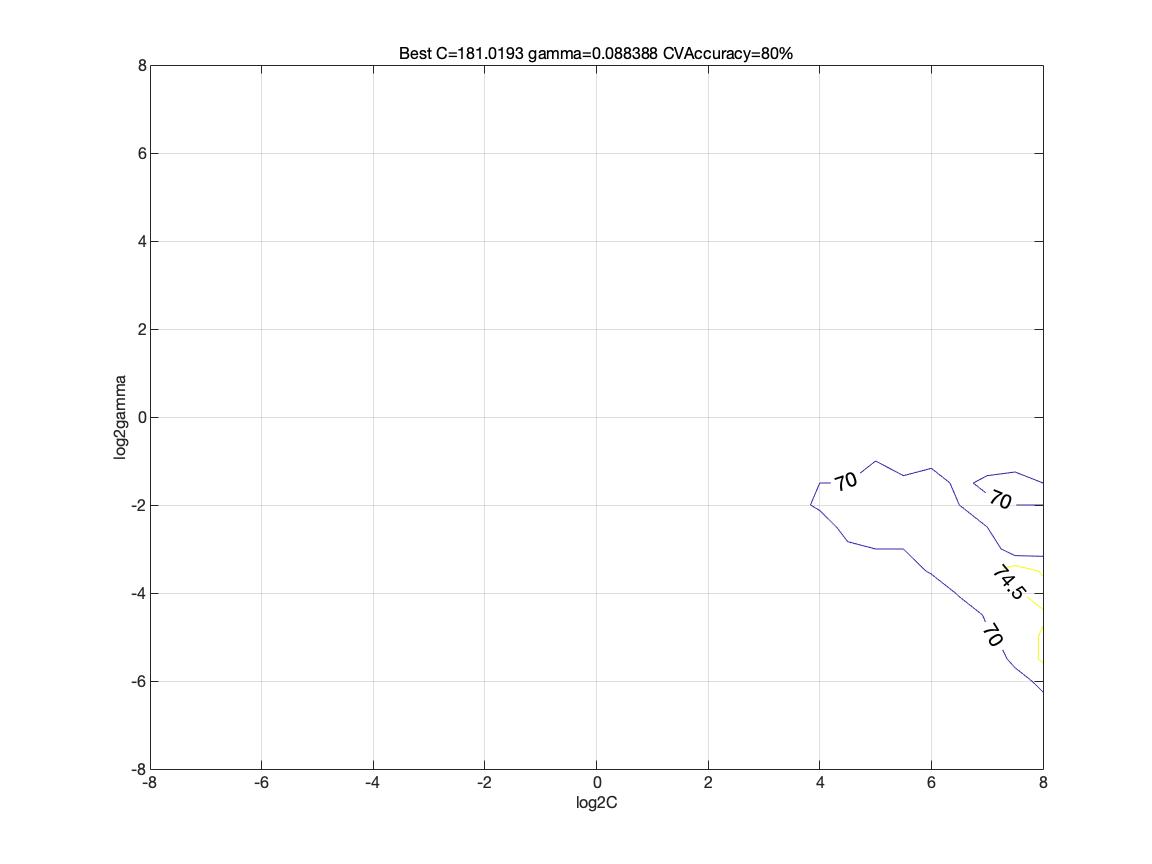


**Figure 2.** CH: 41, the grid searching results of the two RBF parameters used in SVM.


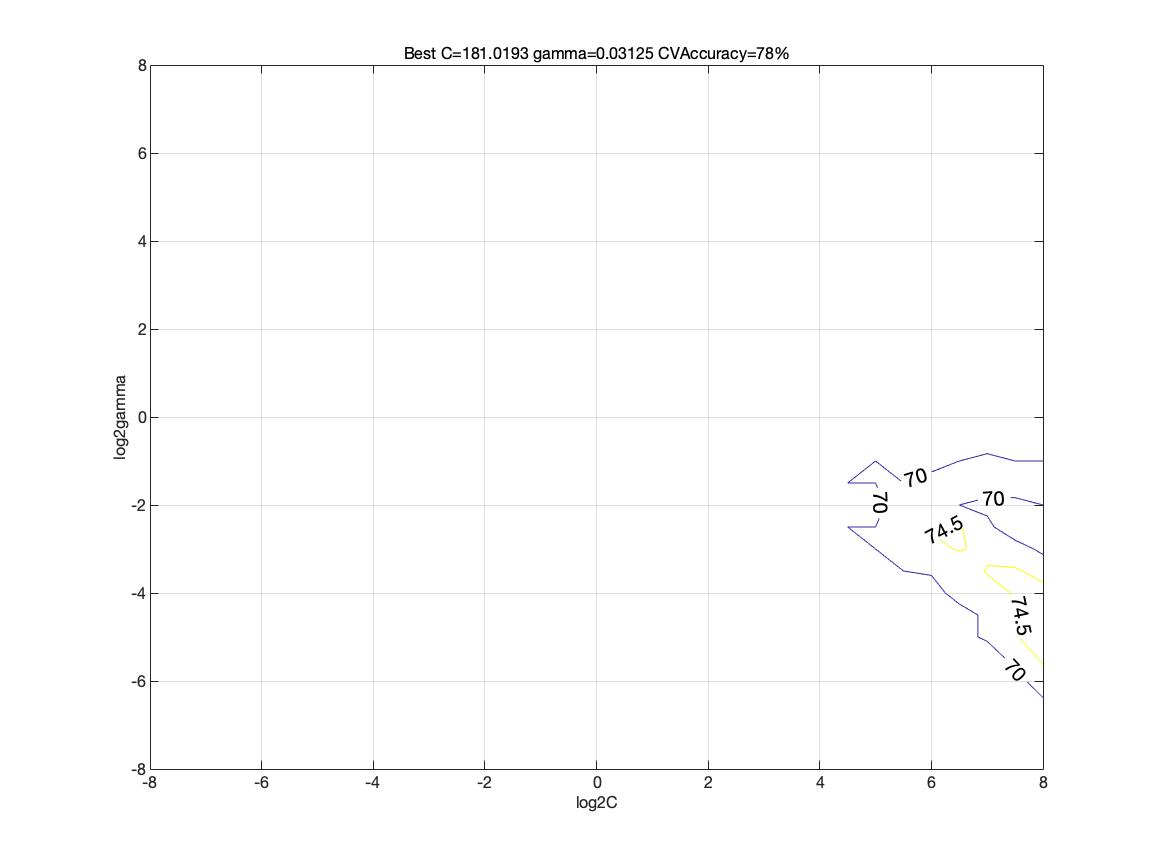


**Figure 3.** CH: 40, the grid searching results of the two RBF parameters used in SVM.


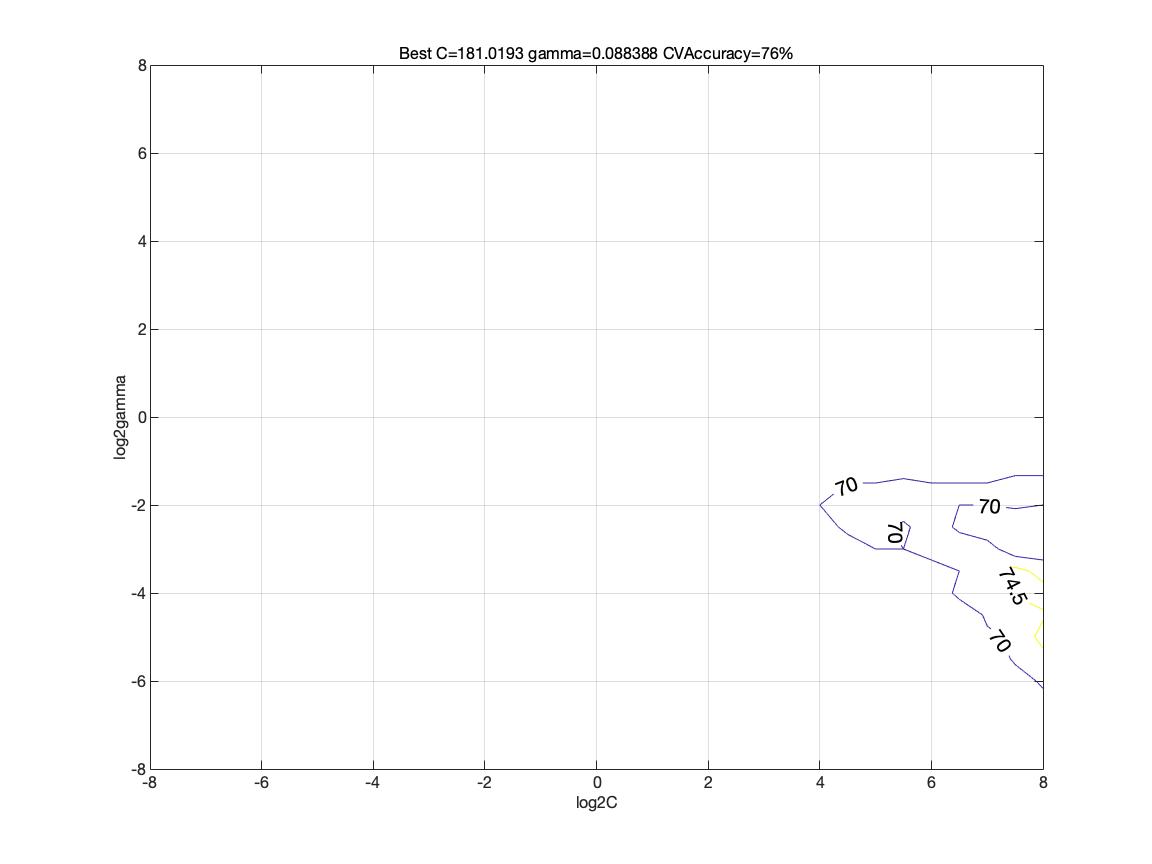


**Figure 4.** CH: 44, the grid searching results of the two RBF parameters used in SVM.


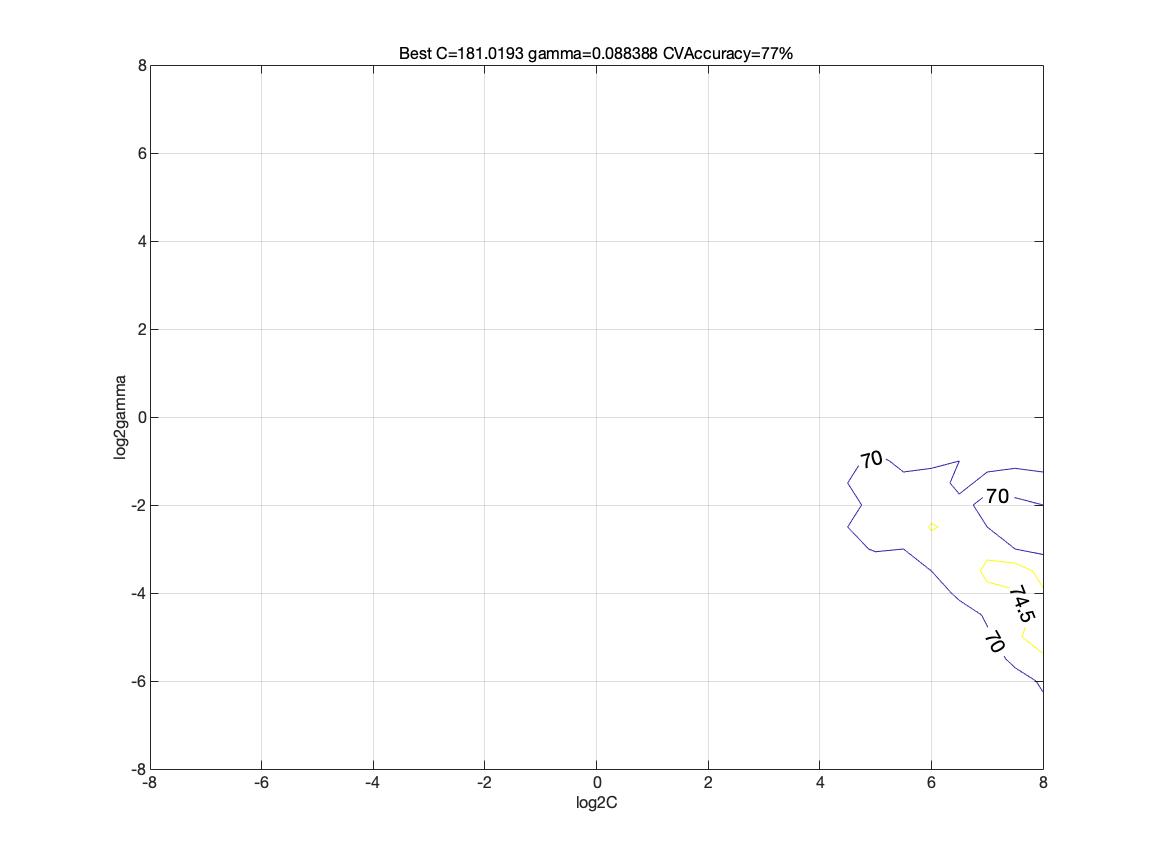


**Figure 5.** CH: 52, the grid searching results of the two RBF parameters used in SVM.


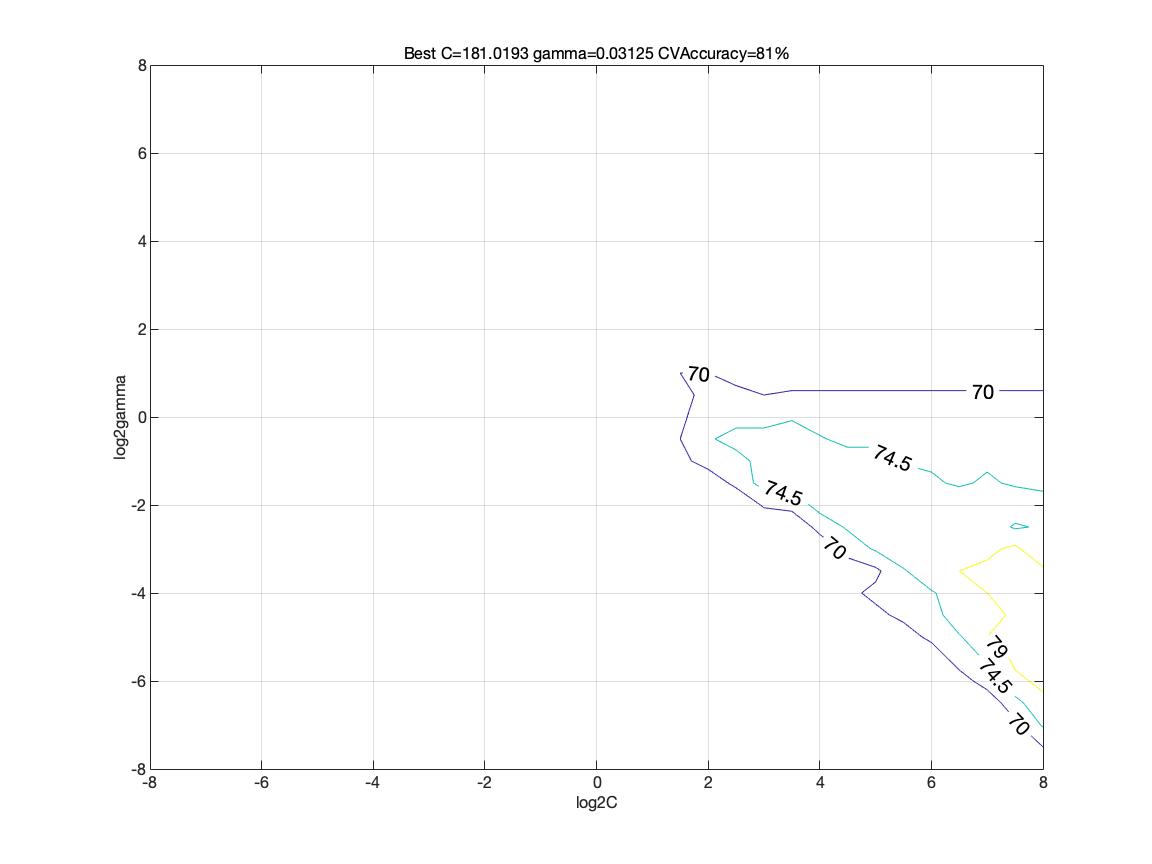


**Figure 6.** 52 classification features, the grid searching results of the two RBF parameters used in SVM.


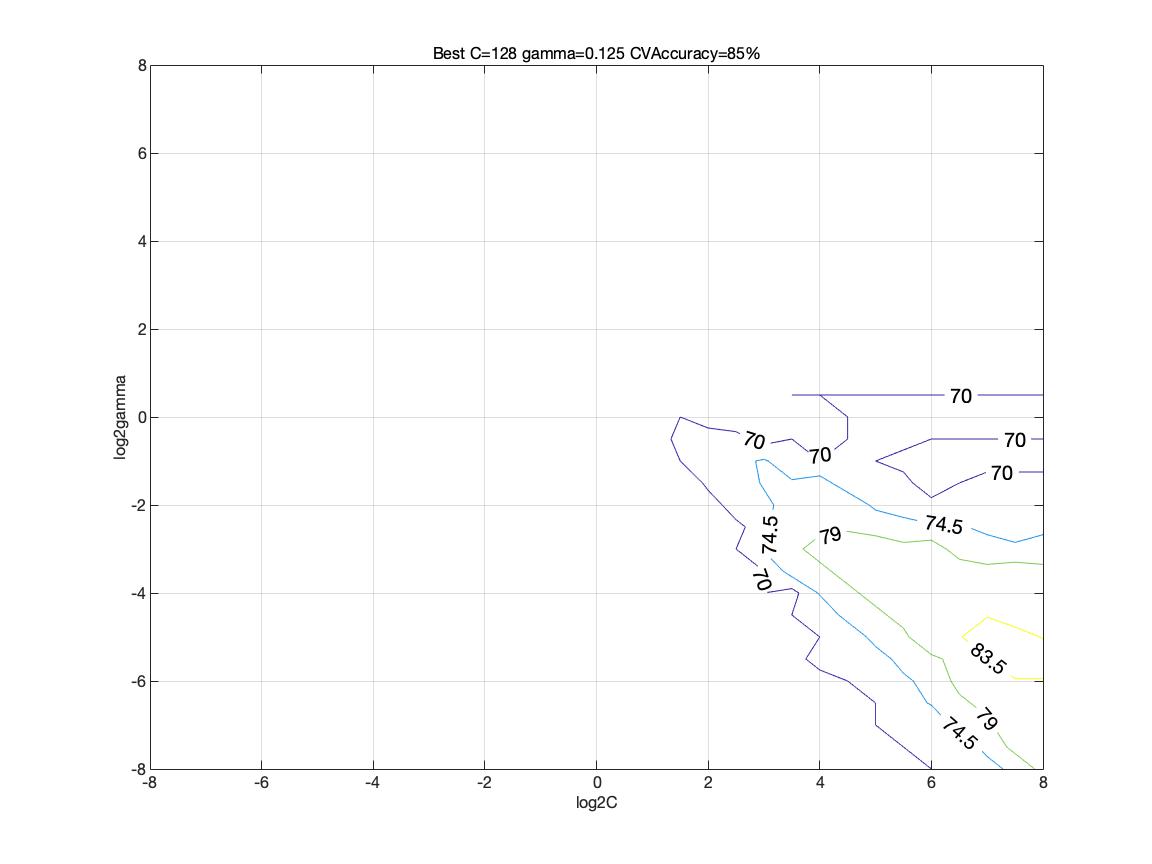


**Figure 7.** LOOCV and PCA, the grid searching results of the two RBF parameters used in SVM.


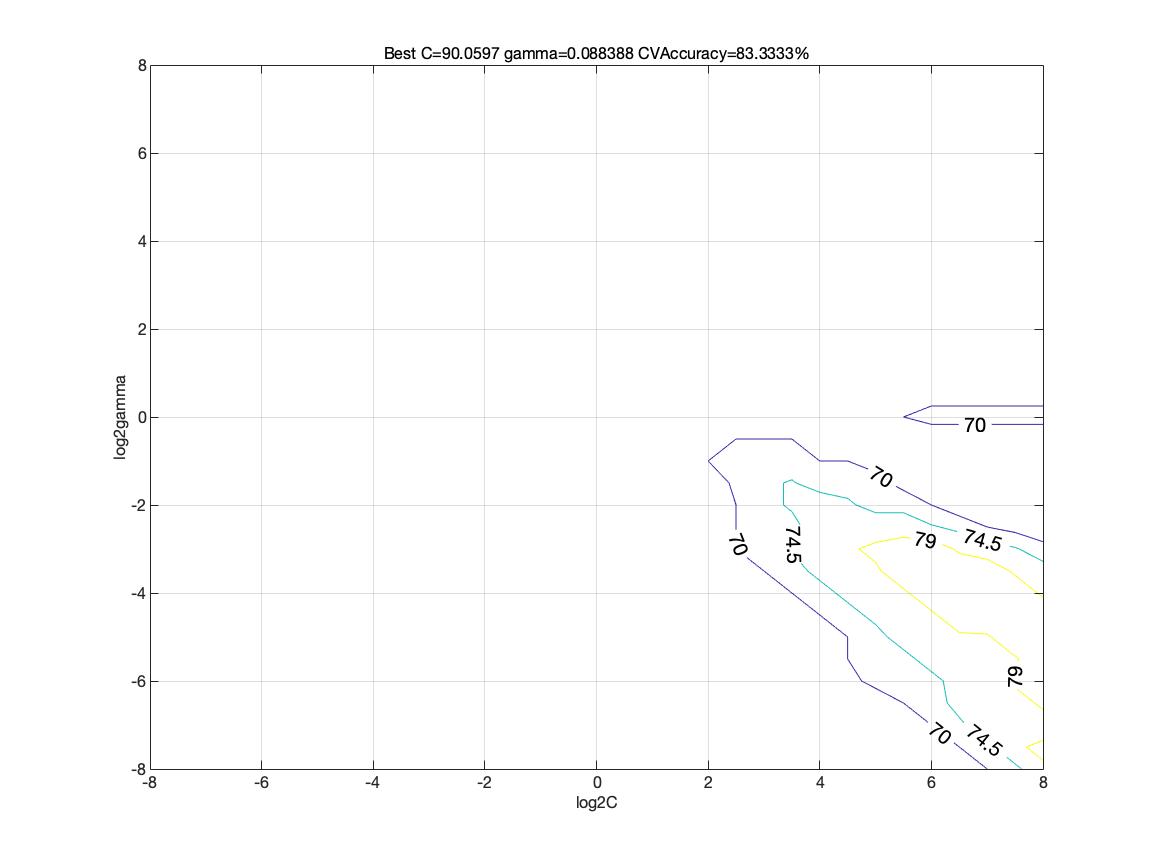


**Figure 8.** LOOCV and KernelPCA, the grid searching results of the two RBF parameters used in SVM.


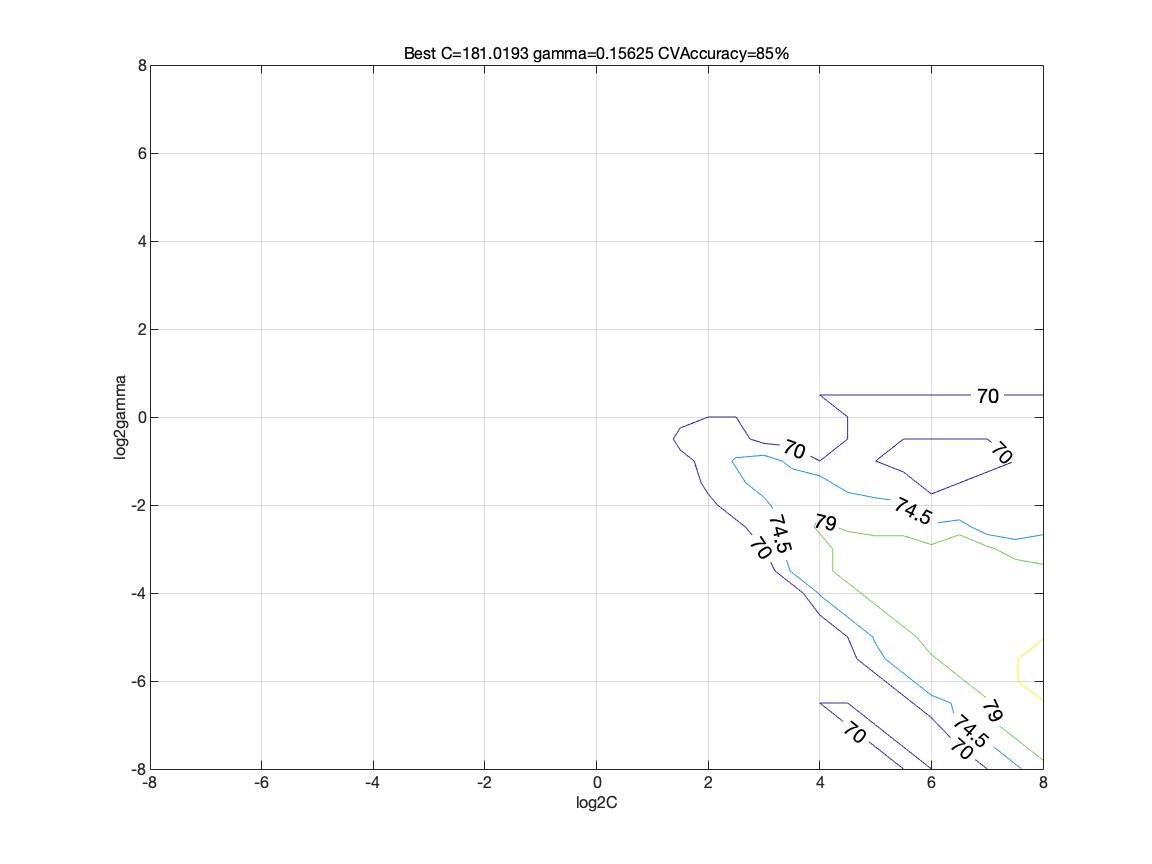


**Figure 9.** LOOCV and SparsePCA, the grid searching results of the two RBF parameters used in SVM.


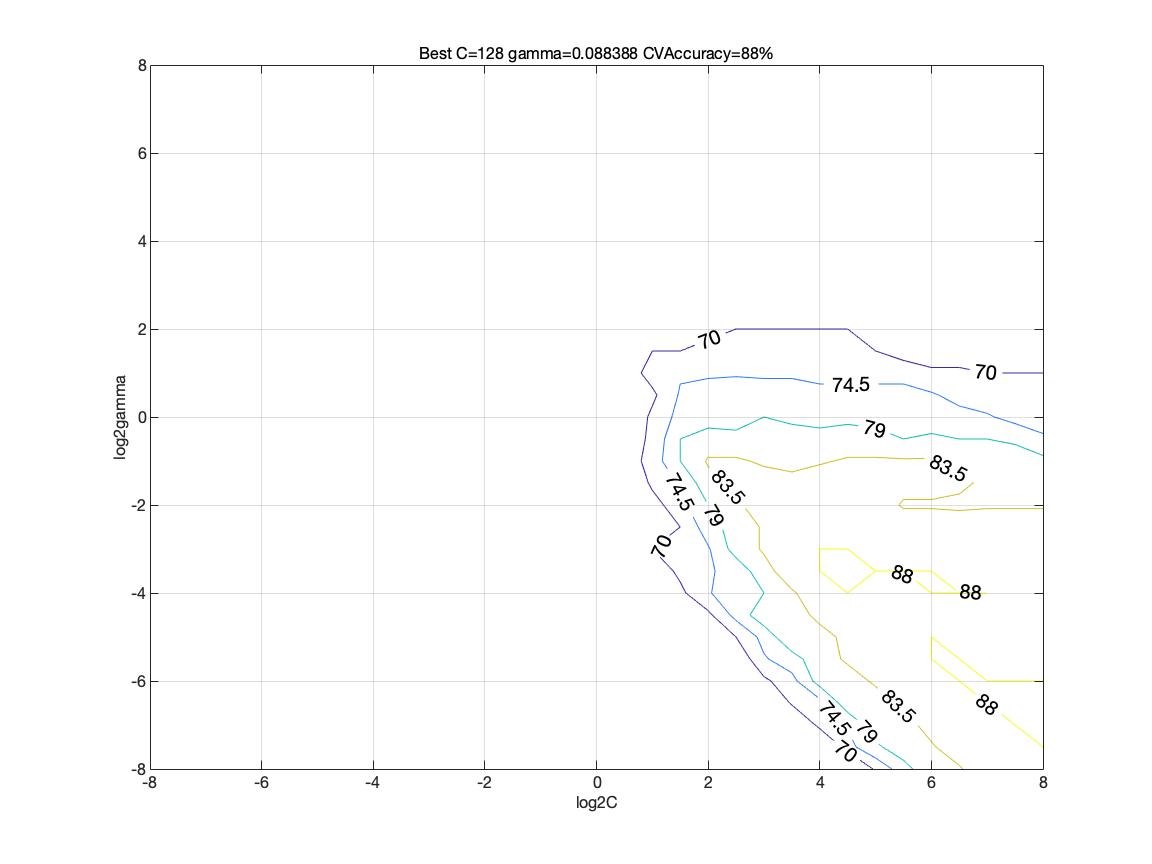


**Figure 10.** 10-fold and PCA, the grid searching results of the two RBF parameters used in SVM.


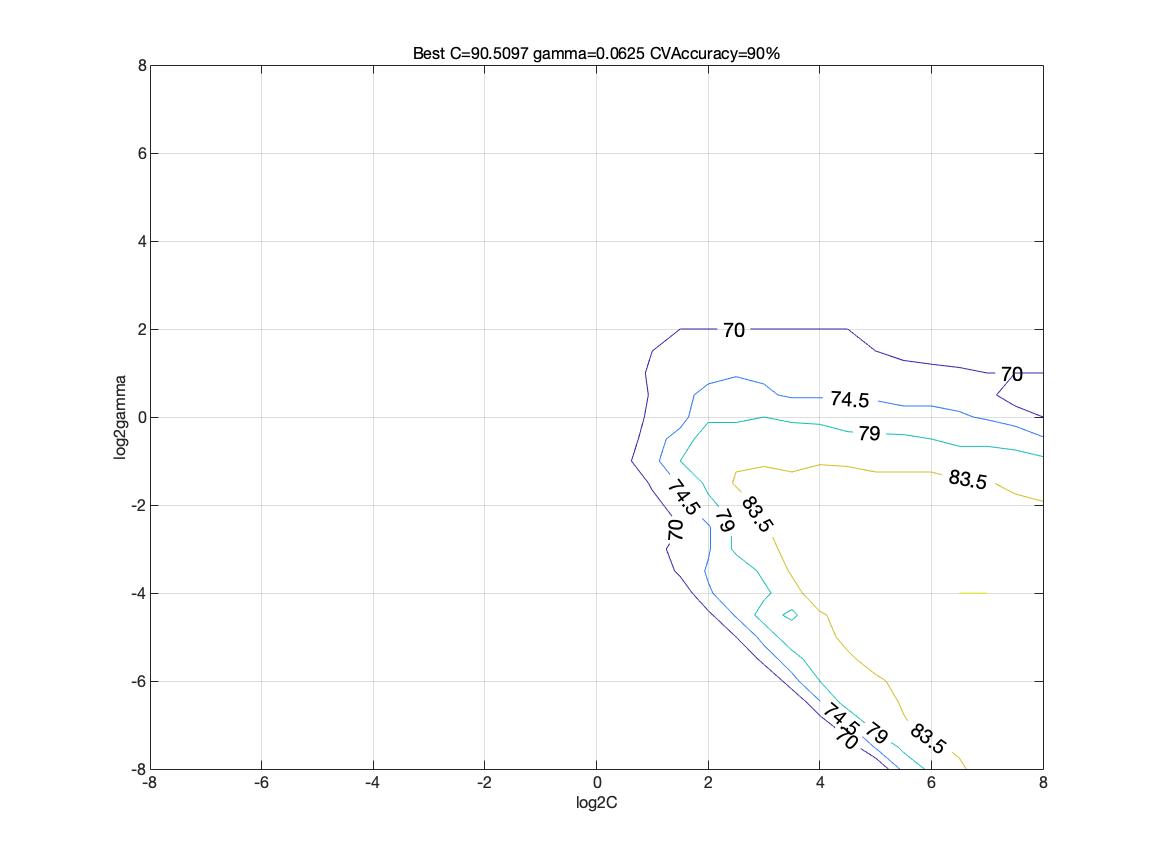


**Figure 11.** 10-fold and KernelPCA, the grid searching results of the two RBF parameters used in SVM.


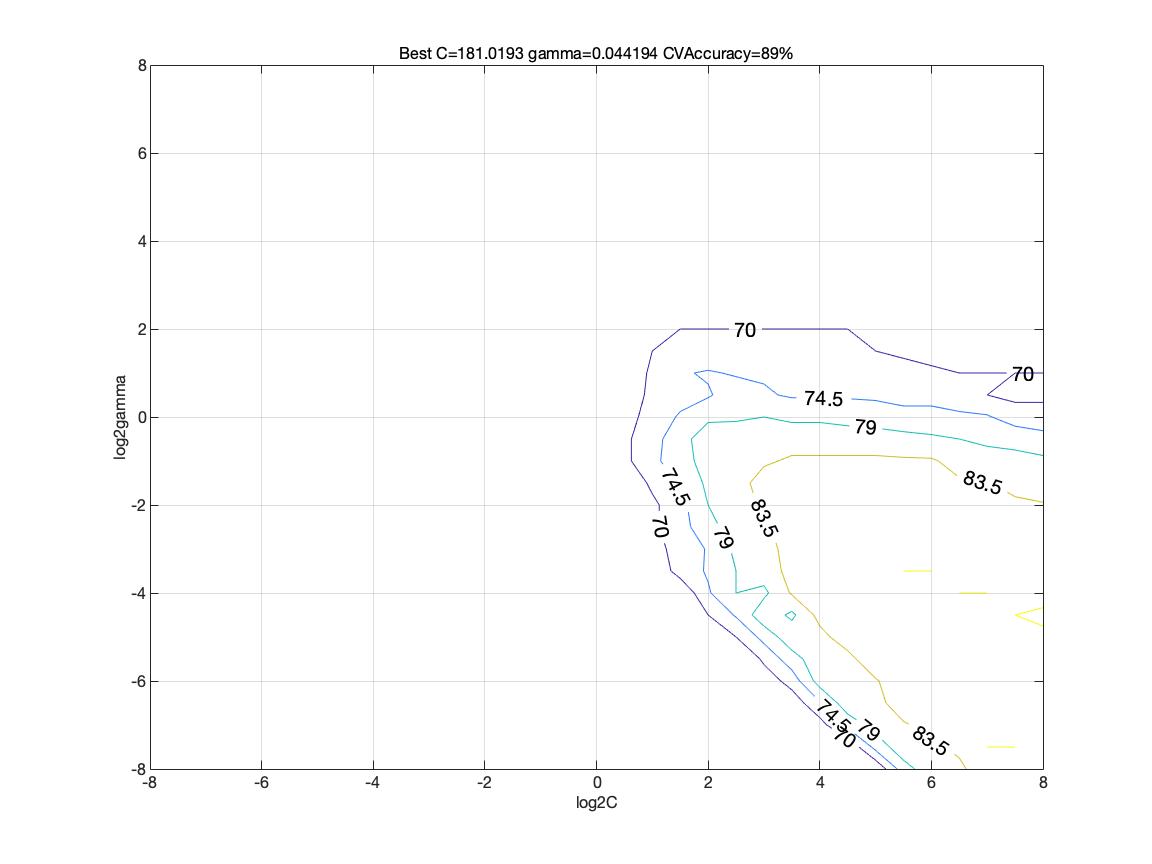


**Figure 12.** 10-fold and SparsePCA, the grid searching results of the two RBF parameters used in SVM.


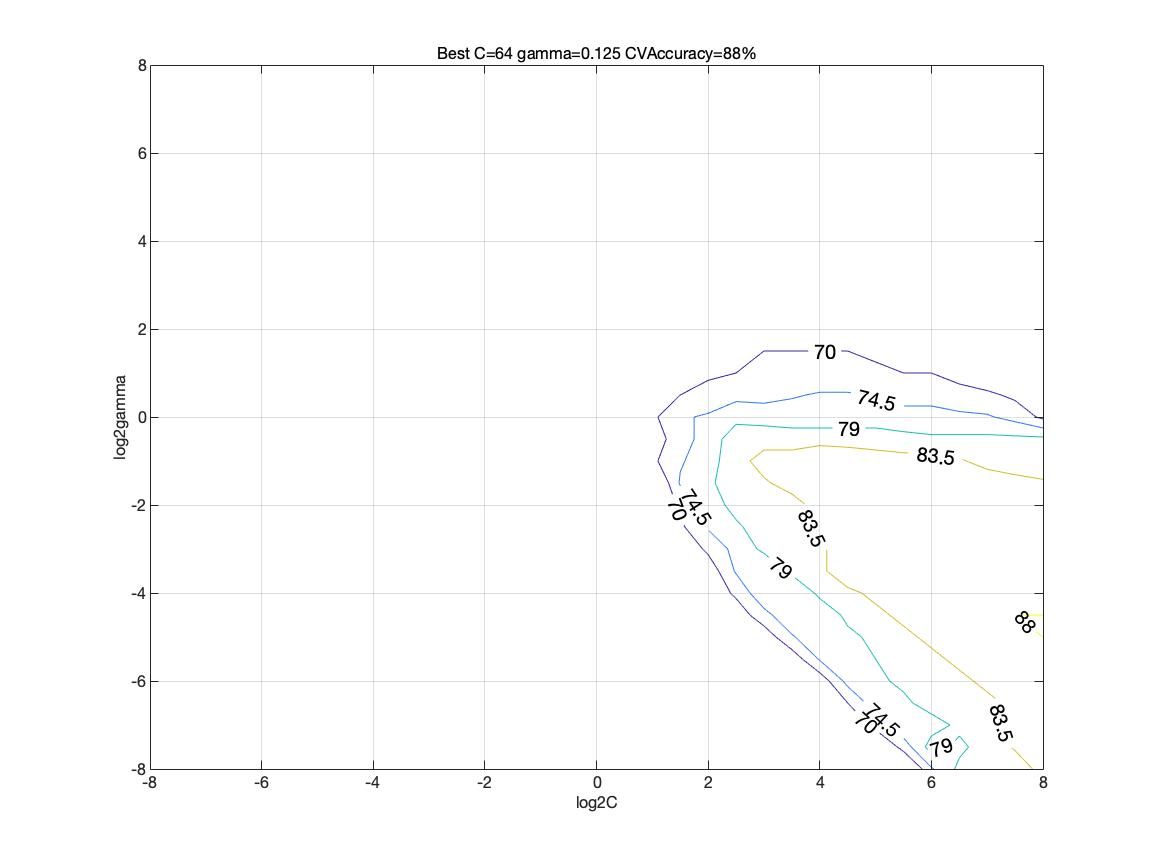


**Figure 13.** 20-fold and PCA, the grid searching results of the two RBF parameters used in SVM.


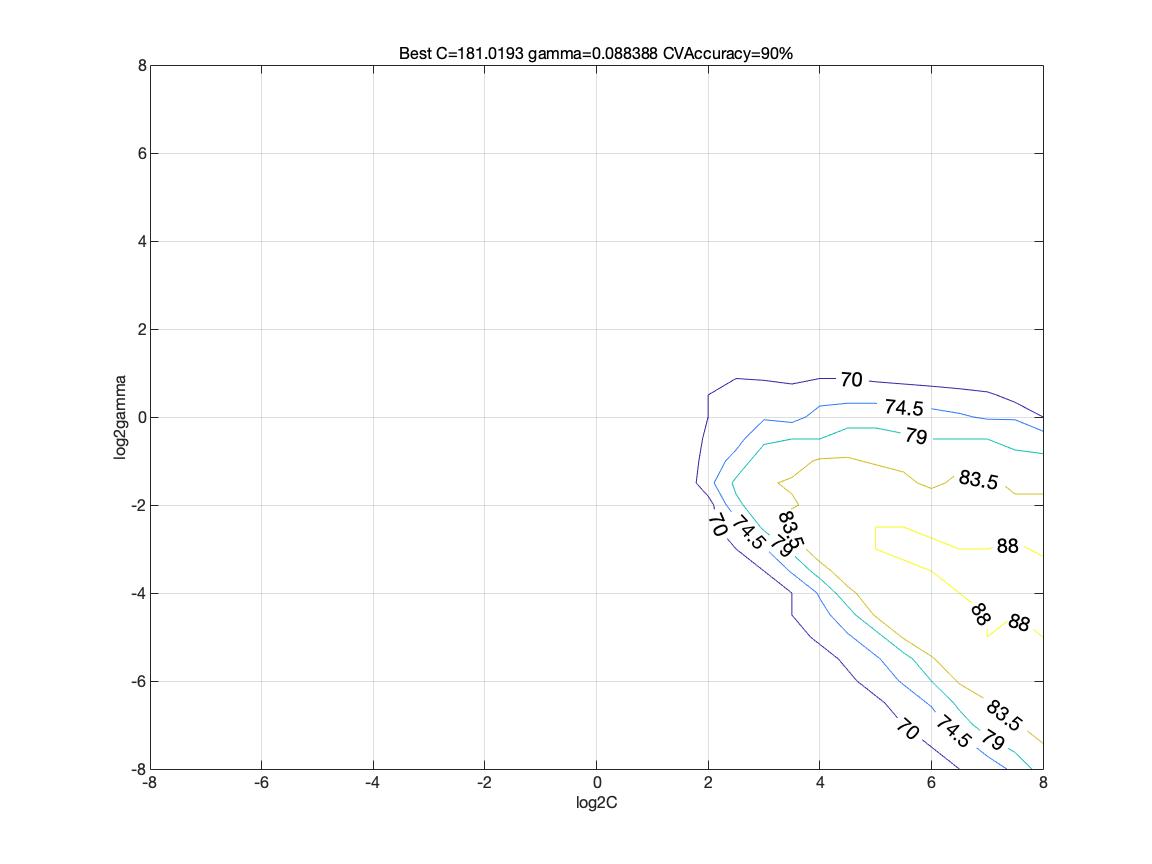


**Figure 14.** 20-fold and KernelPCA, the grid searching results of the two RBF parameters used in SVM.


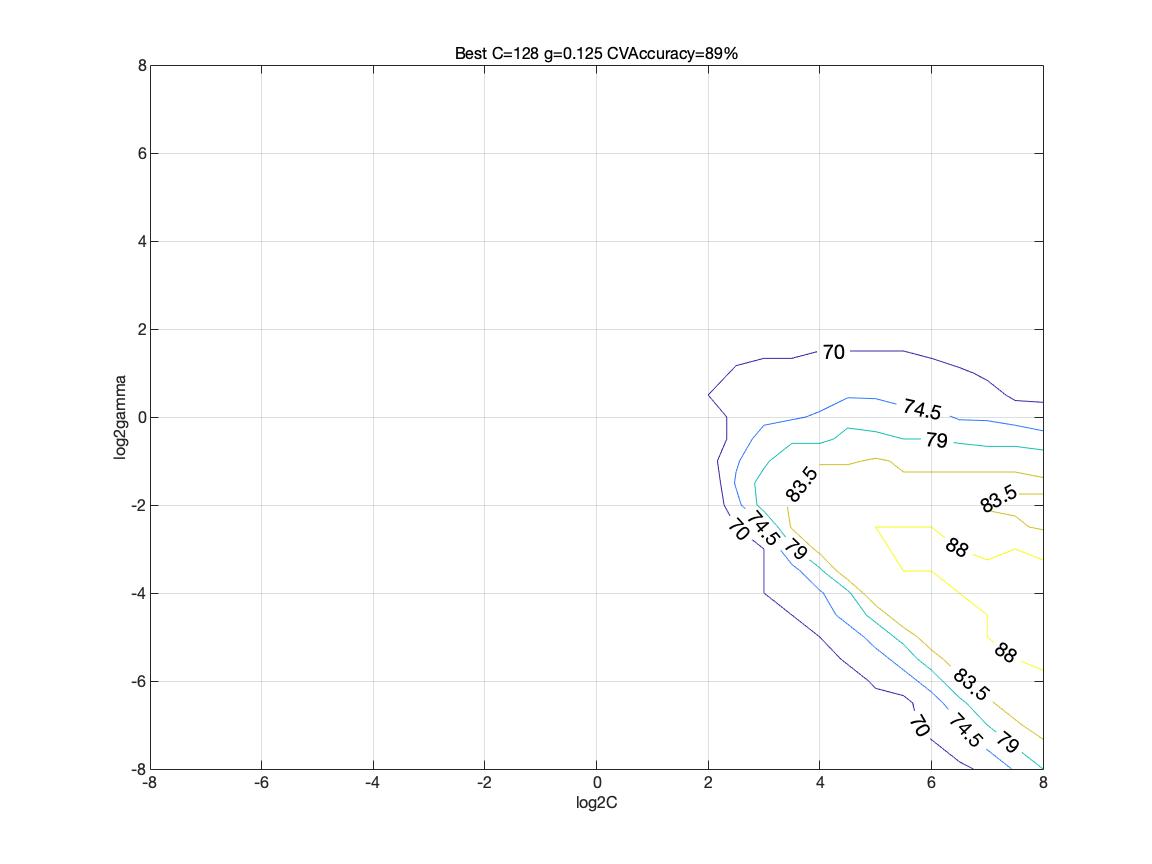


**Figure 15.** 20-fold and SparsePCA, the grid searching results of the two RBF parameters used in SVM.


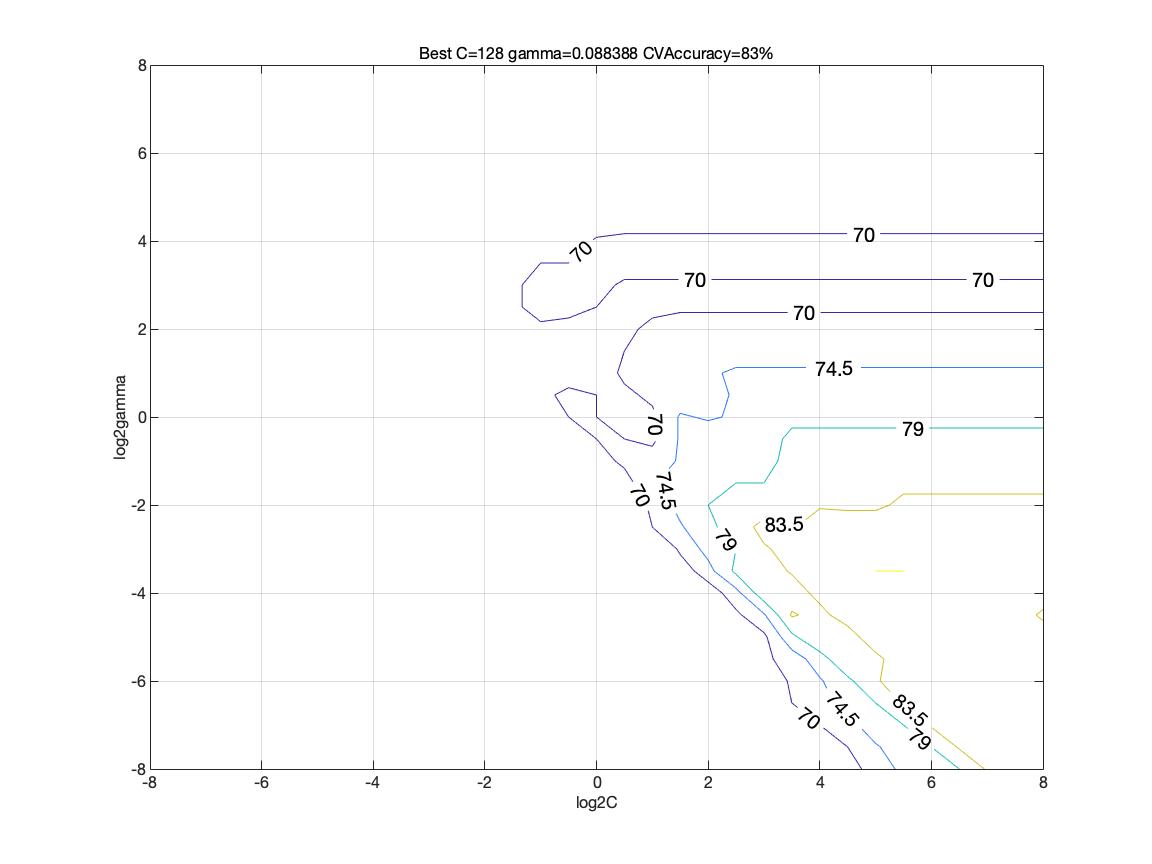


**Figure 16.** CH: 40 and 41, the grid searching results of the two RBF parameters used in SVM.


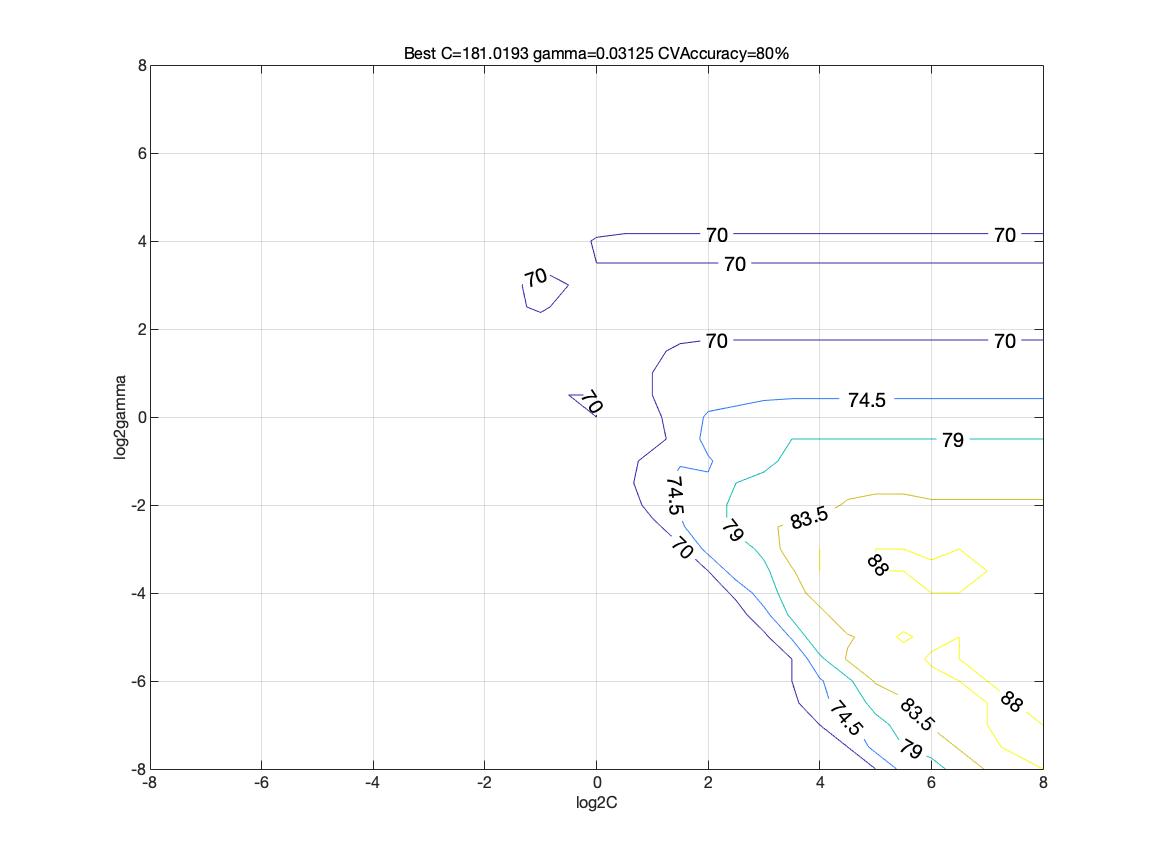


**Figure 17.** CH: 40 and 44, the grid searching results of the two RBF parameters used in SVM.


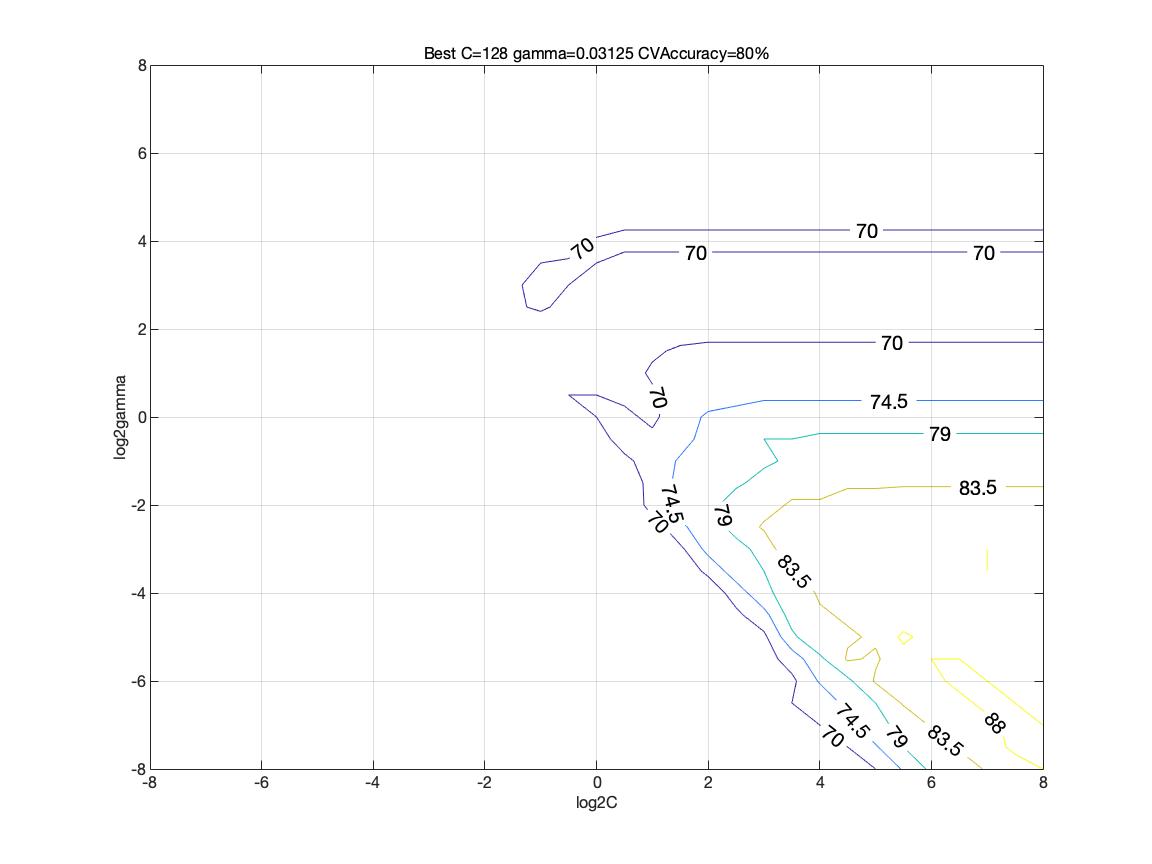


**Figure 18.** CH: 40 and 50, the grid searching results of the two RBF parameters used in SVM.


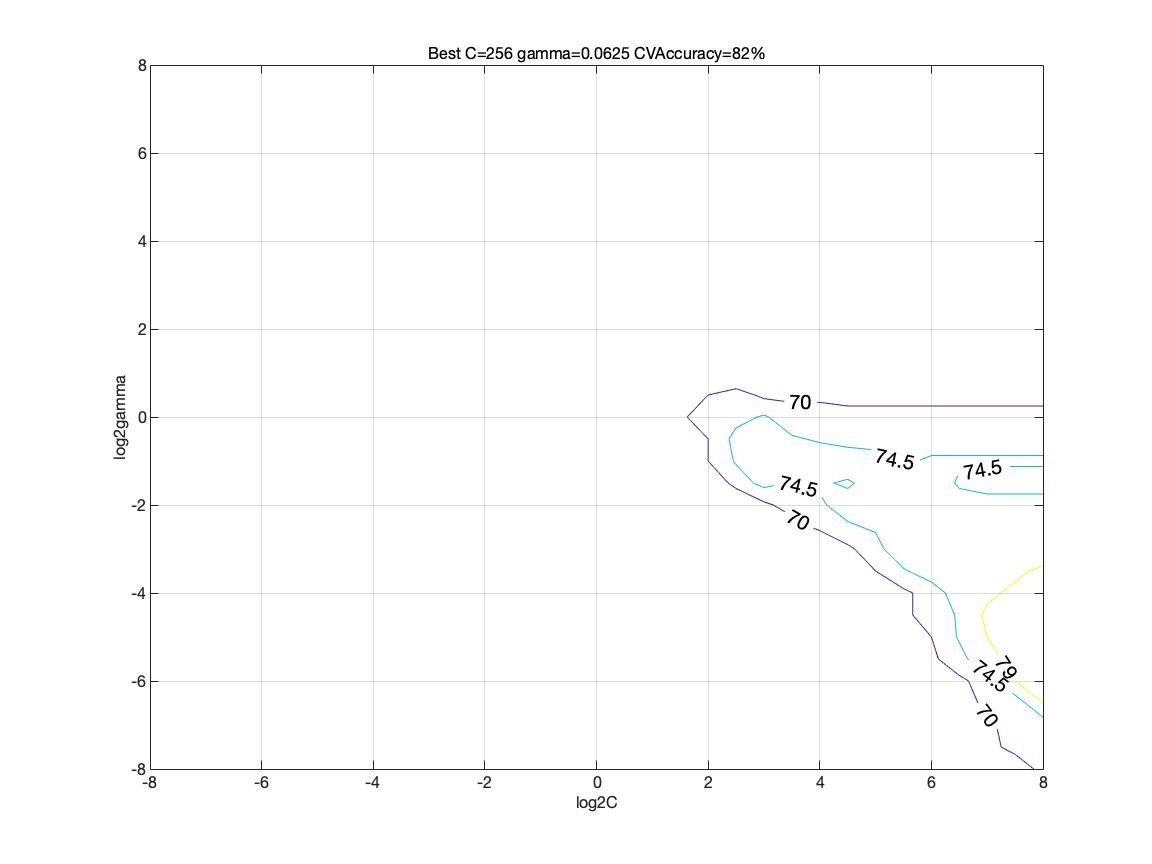


**Figure 19.** CH: 40 and 52, the grid searching results of the two RBF parameters used in SVM.


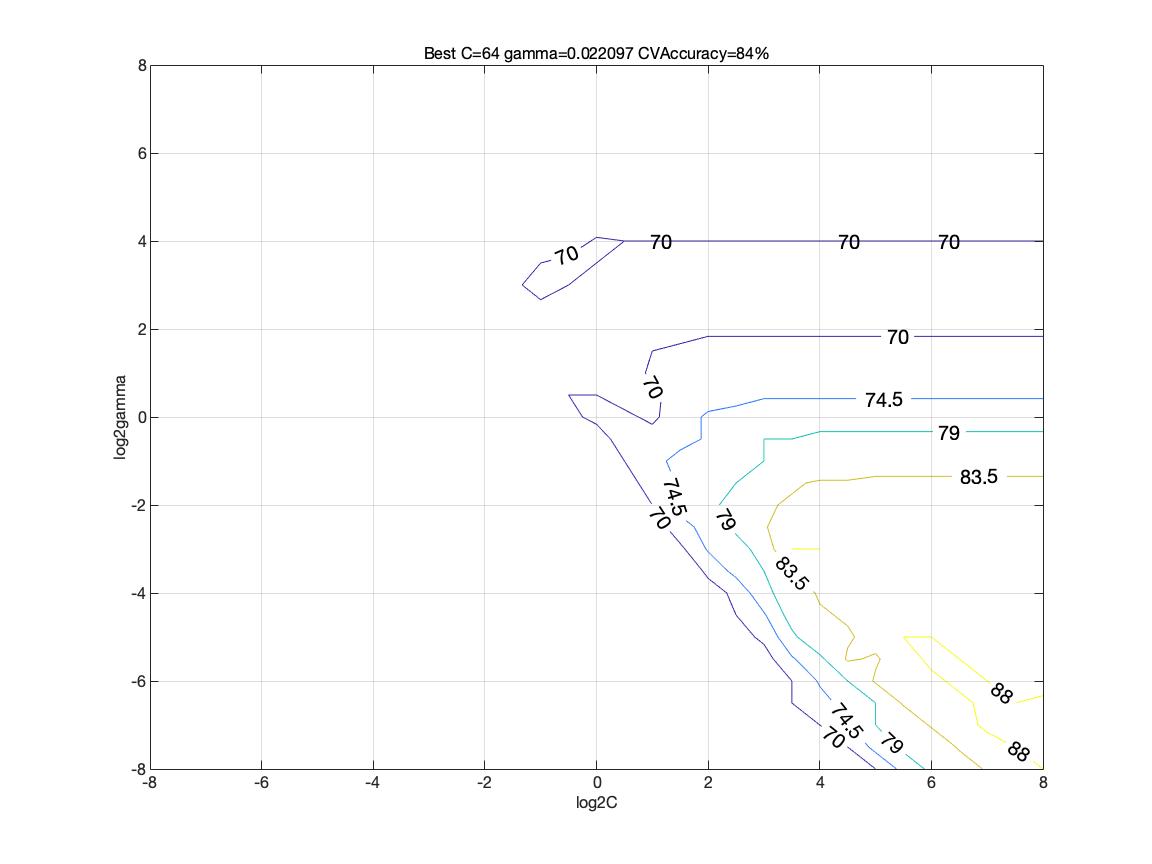


**Figure 20.** CH: 41 and 44, the grid searching results of the two RBF parameters used in SVM.


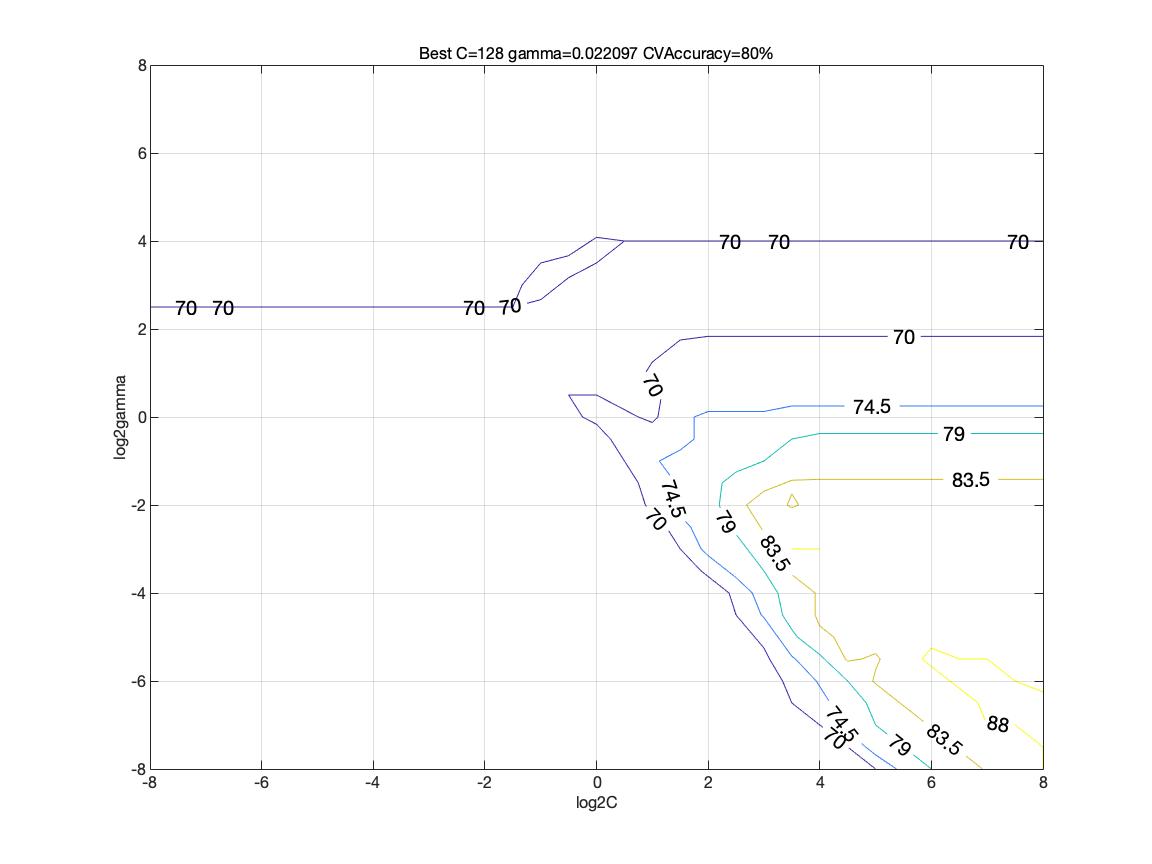


**Figure 21.** CH: 41 and 50, the grid searching results of the two RBF parameters used in SVM.


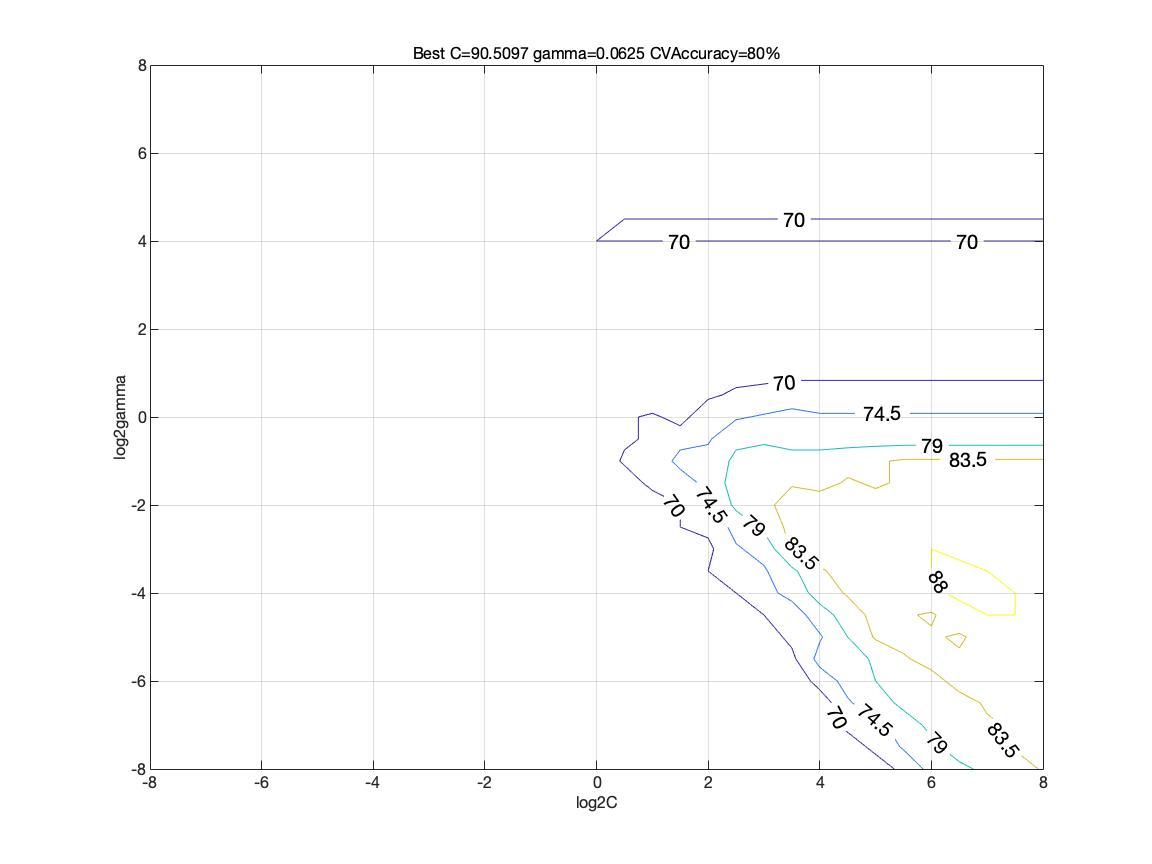


**Figure 22.** CH: 41 and 52, the grid searching results of the two RBF parameters used in SVM.


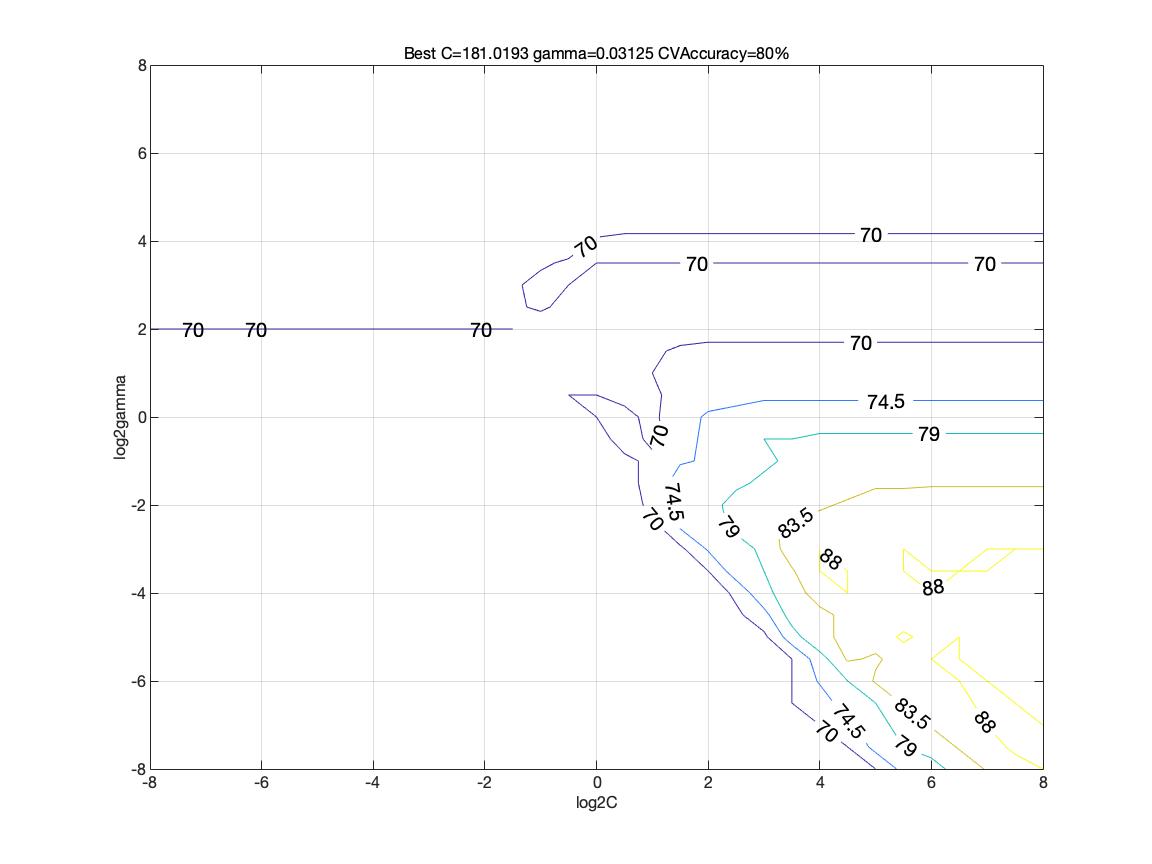


**Figure 23.** CH: 44 and 50, the grid searching results of the two RBF parameters used in SVM.


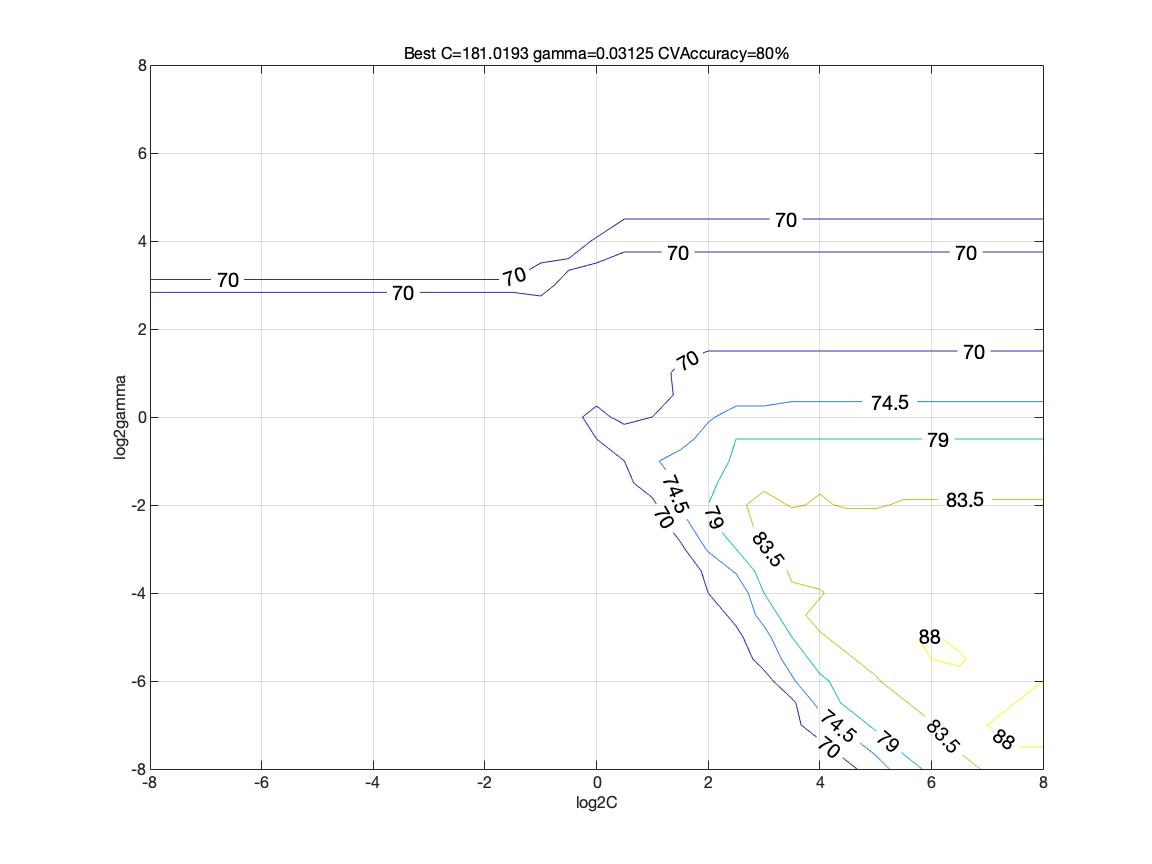


**Figure 24.** CH: 44 and 52, the grid searching results of the two RBF parameters used in SVM.


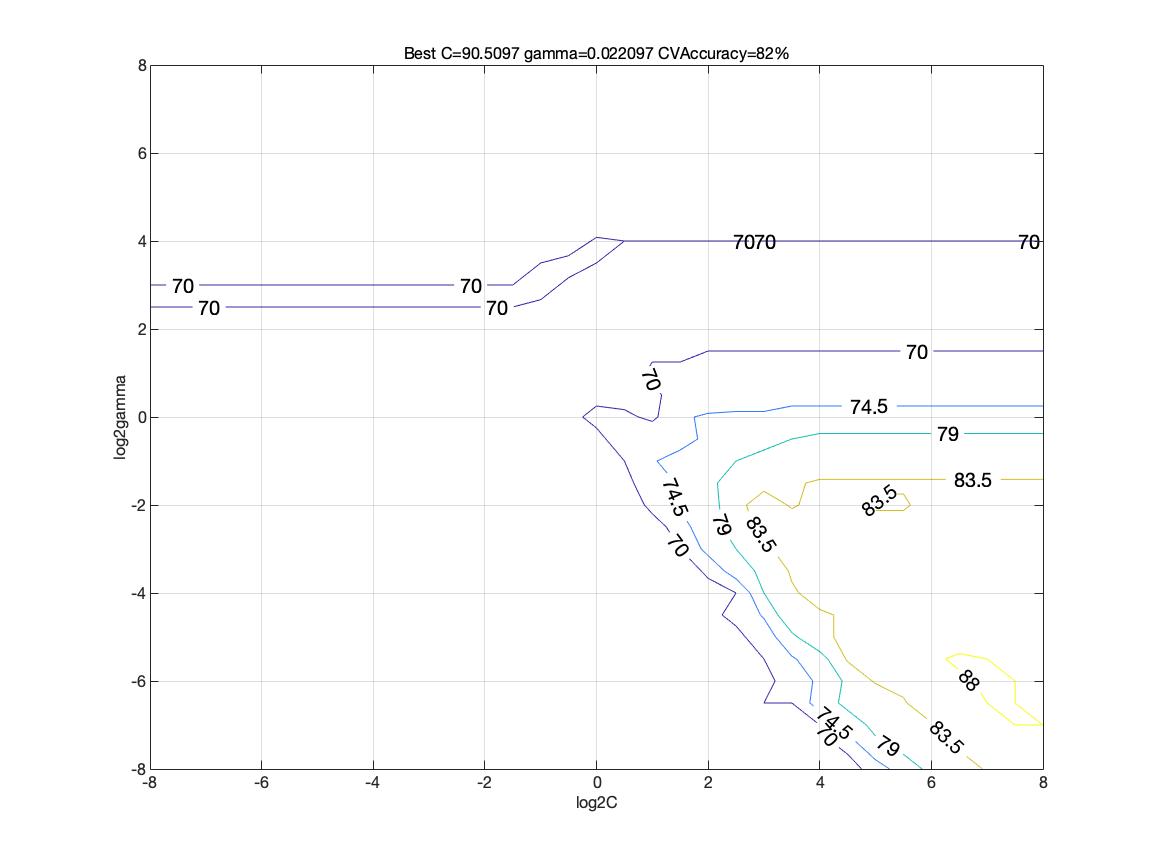


**Figure 25.** CH: 50 and 52, the grid searching results of the two RBF parameters used in SVM.


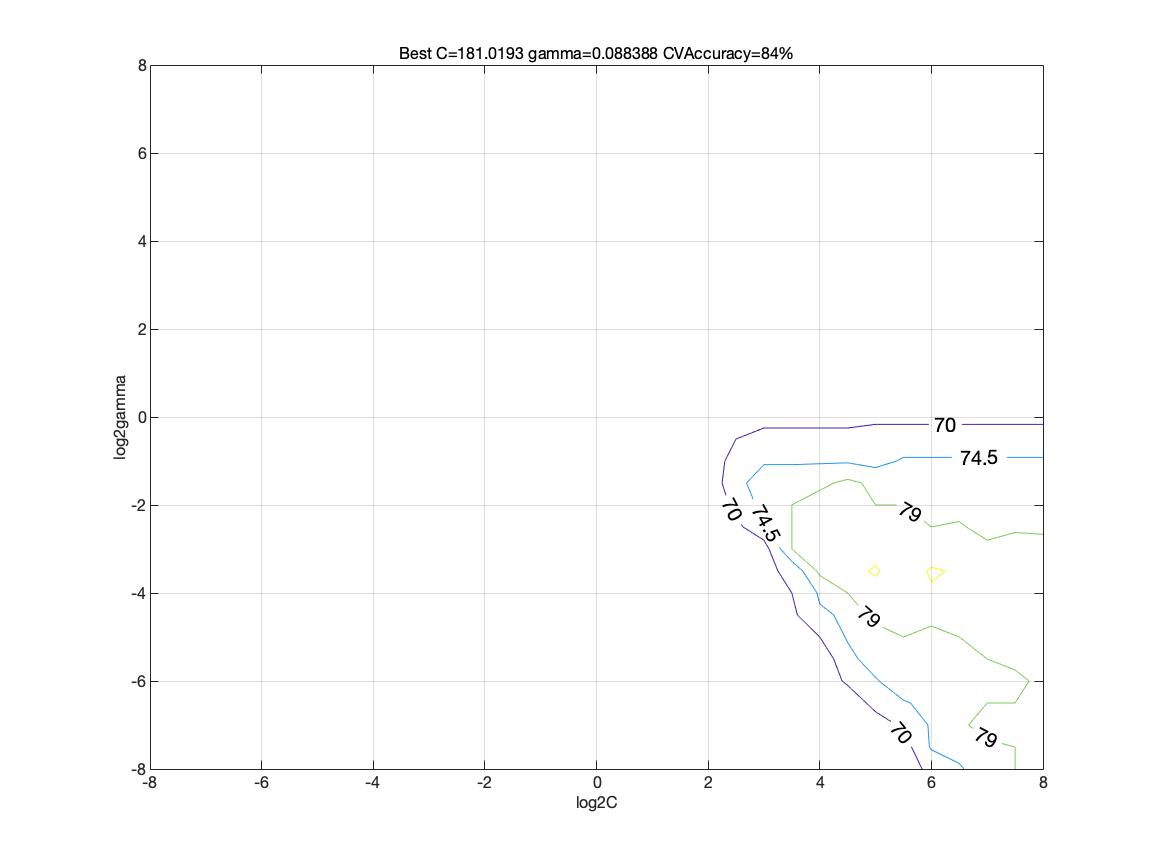


**Figure 26.** CH: 40, 41 and 44, the grid searching results of the two RBF parameters used in SVM.


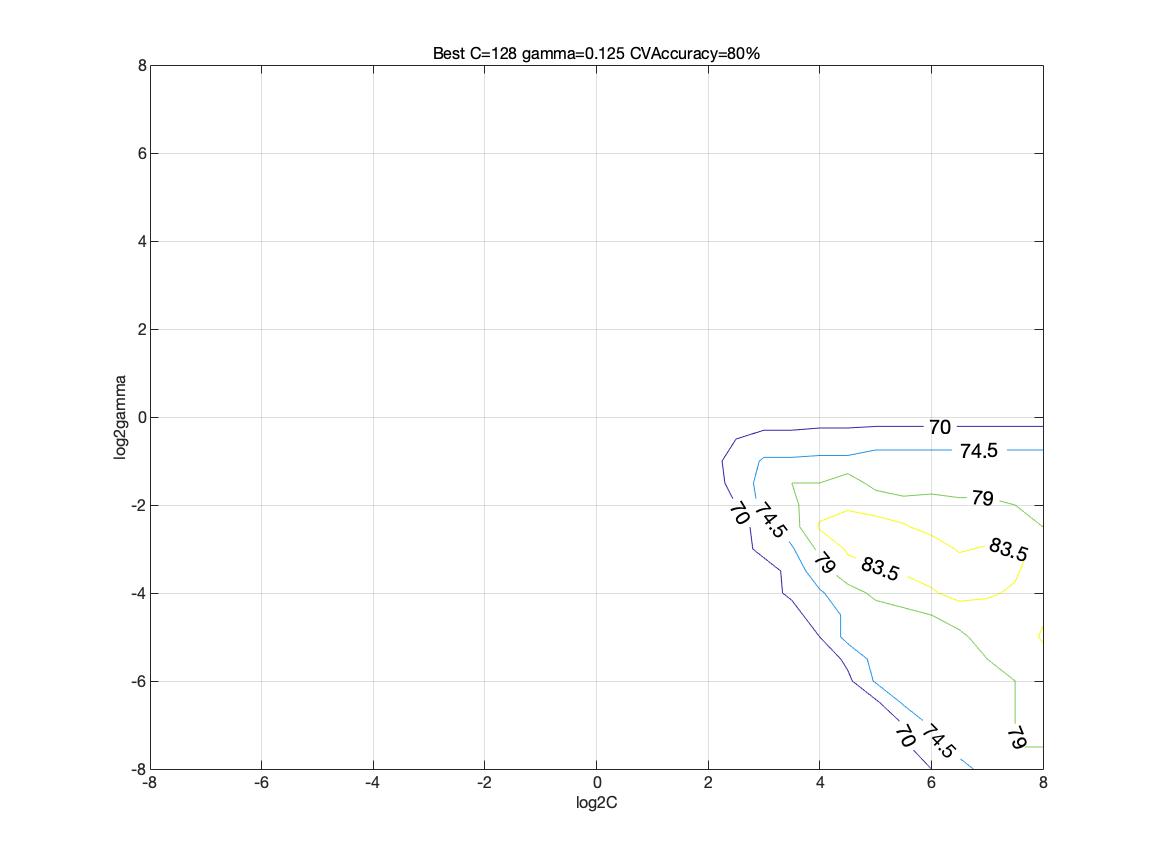


**Figure 27.** CH: 40, 41 and 50, the grid searching results of the two RBF parameters used in SVM.


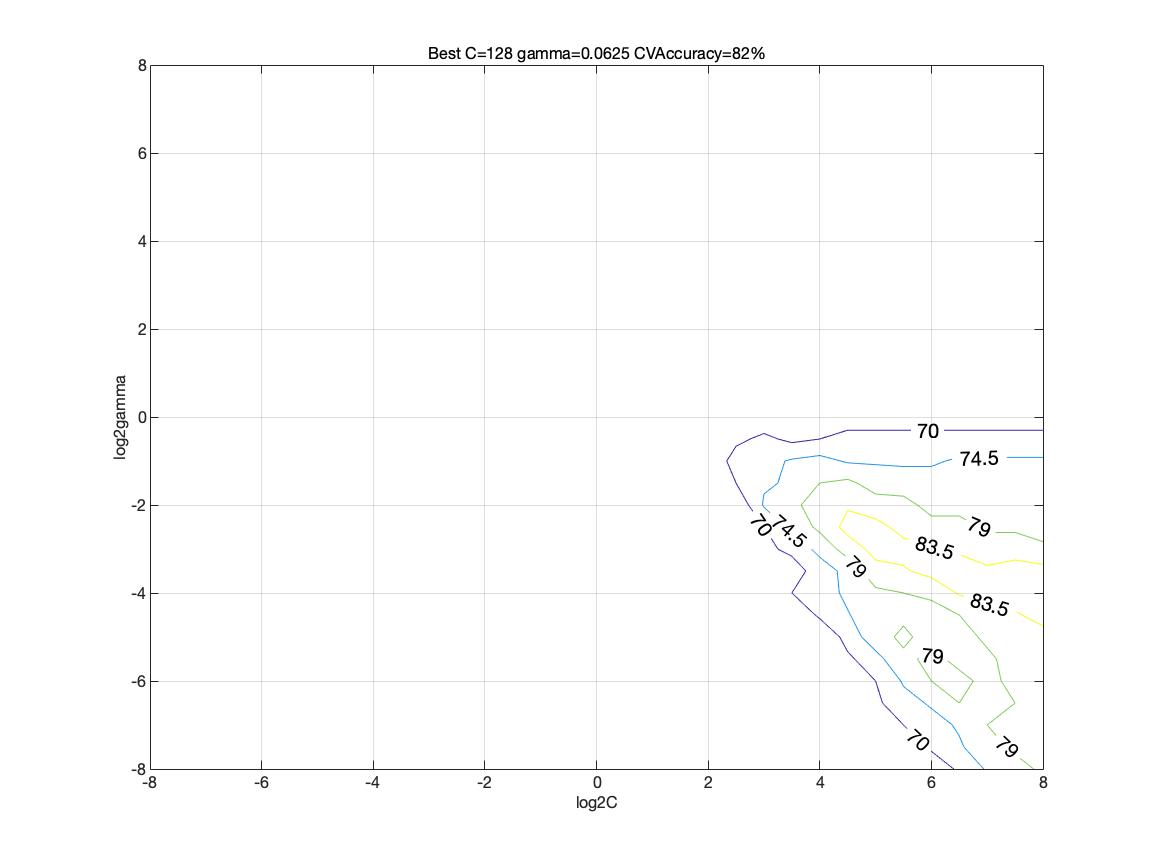


**Figure 28.** CH: 40, 41 and 52, the grid searching results of the two RBF parameters used in SVM.


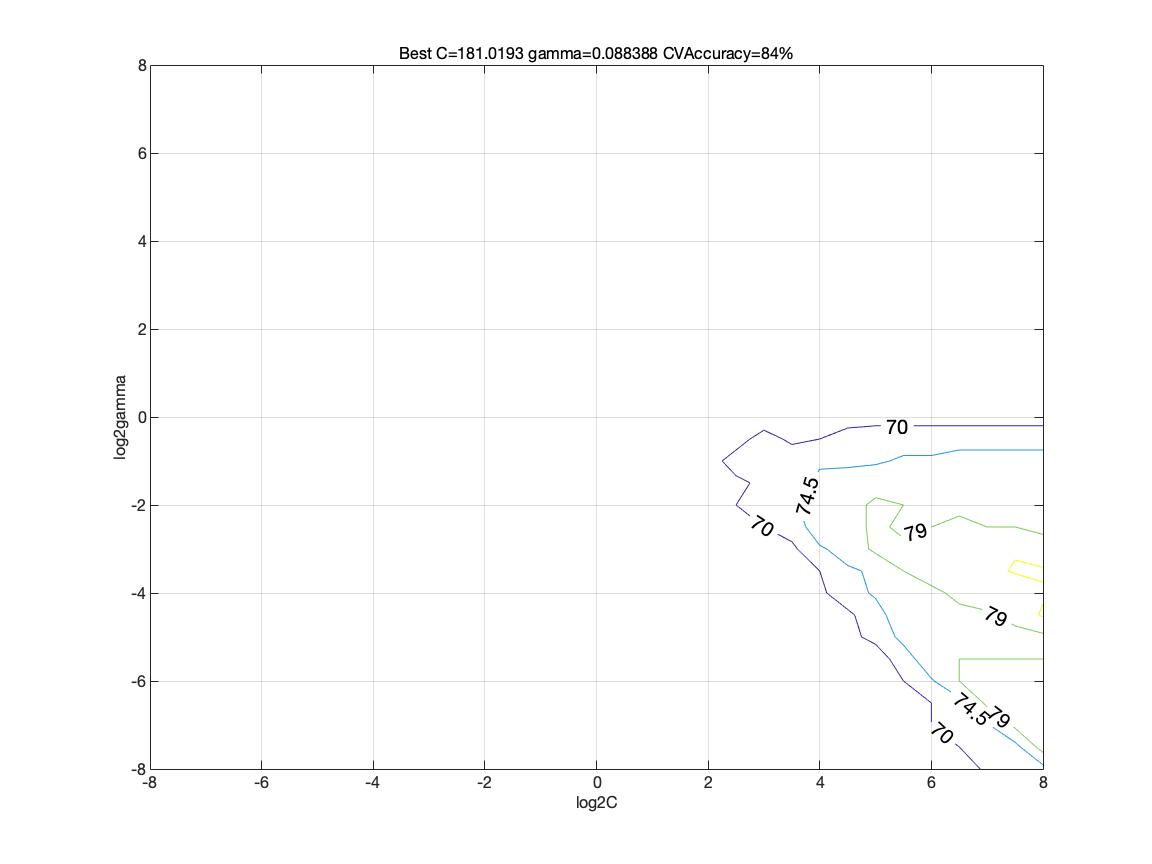


**Figure 29.** CH: 40, 44 and 50, the grid searching results of the two RBF parameters used in SVM.


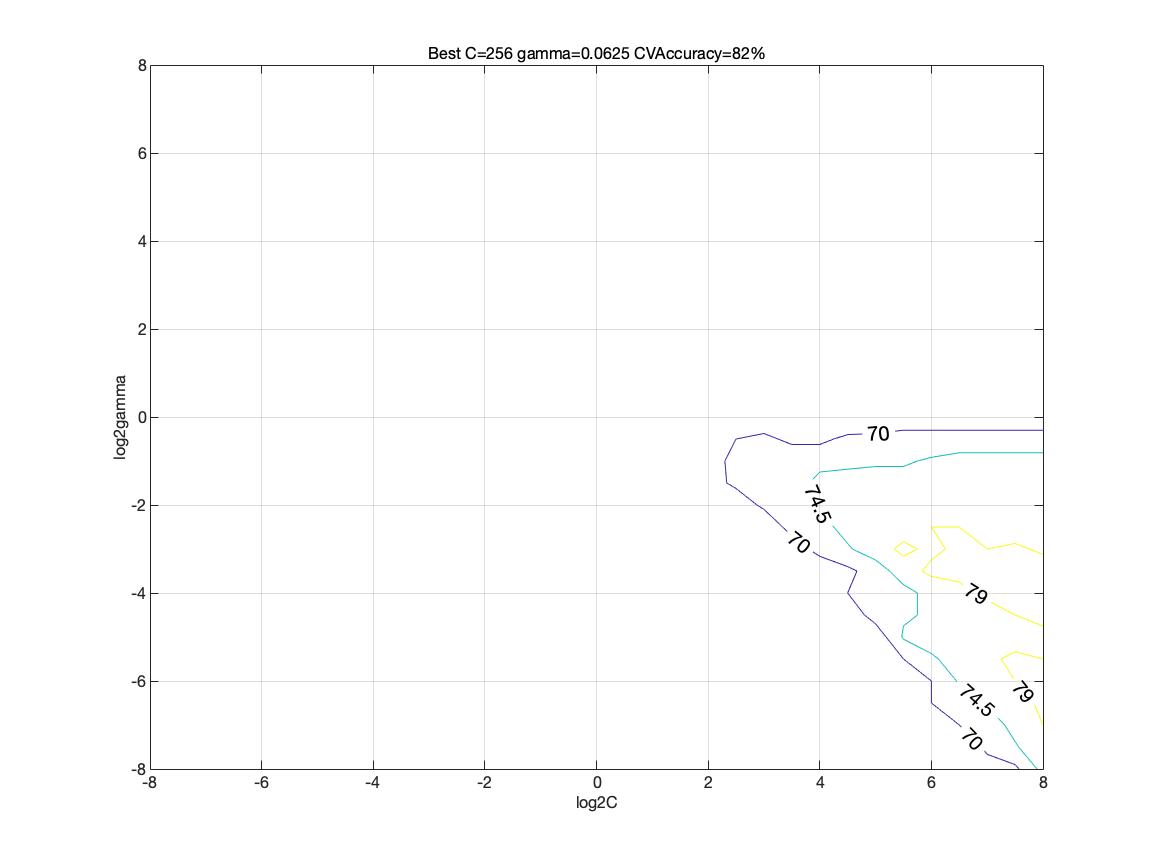


**Figure 30.** CH: 40, 44 and 52, the grid searching results of the two RBF parameters used in SVM.


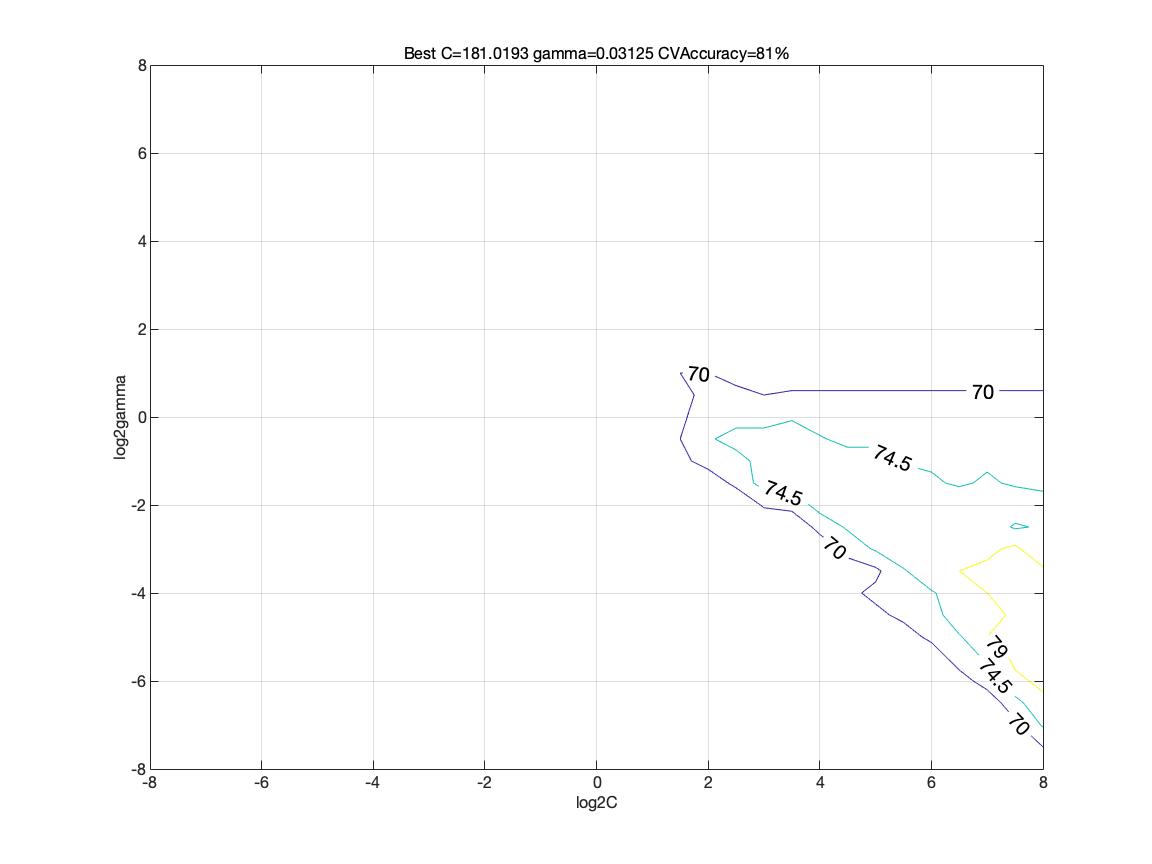


**Figure 31.** CH: 40, 50 and 52, the grid searching results of the two RBF parameters used in SVM.


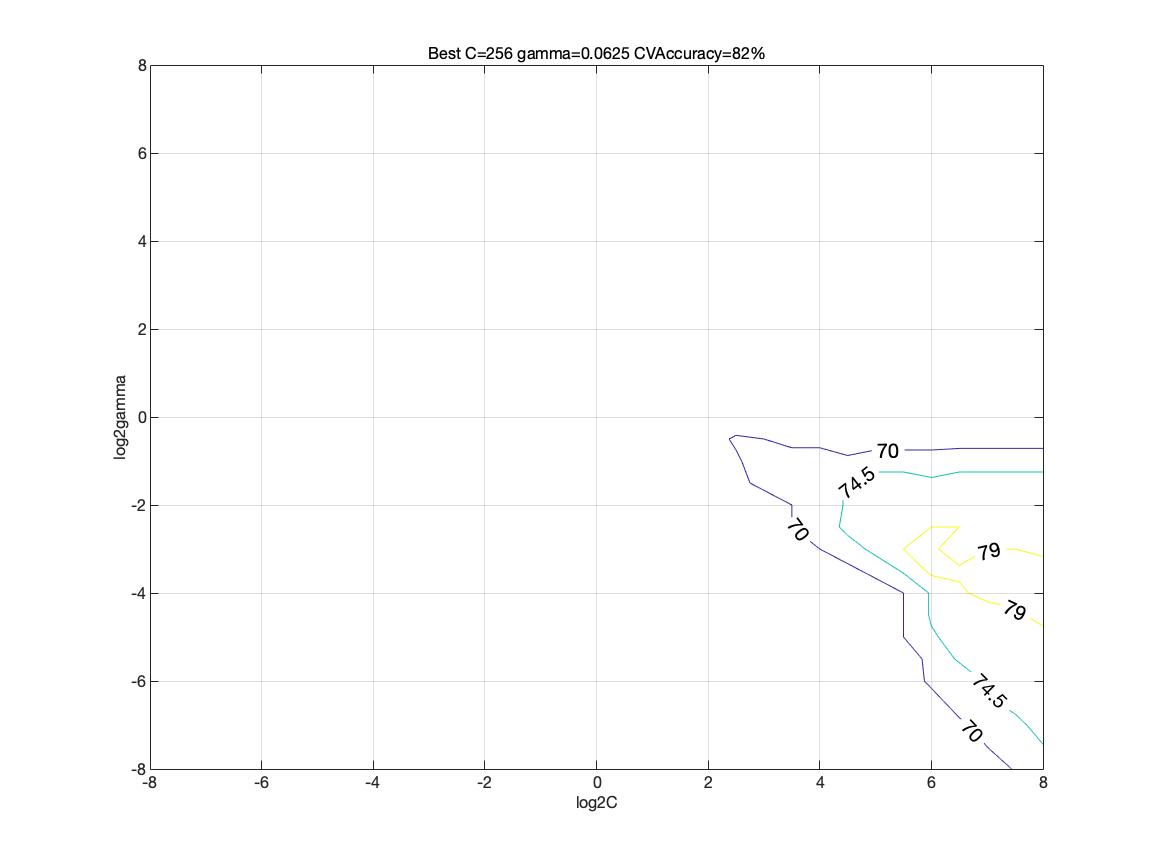


**Figure 32.** CH: 41, 44 and 50, the grid searching results of the two RBF parameters used in SVM.


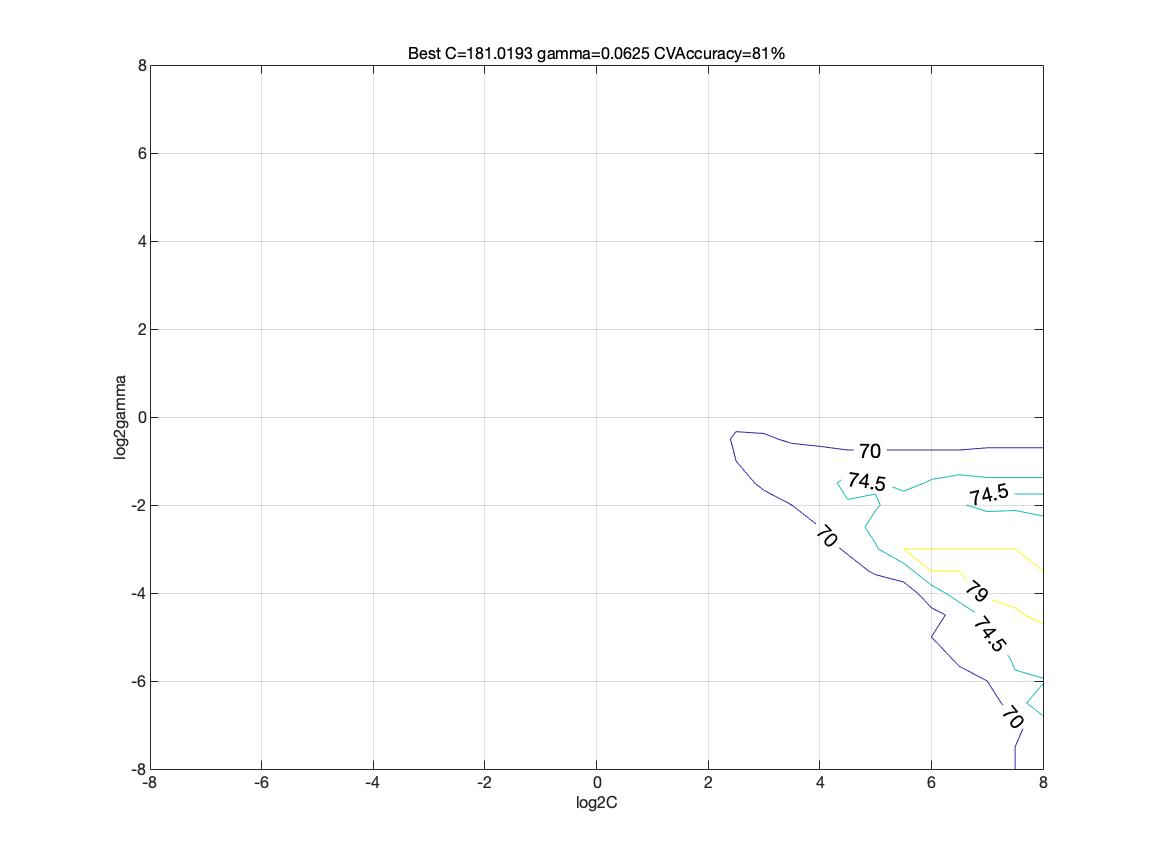


**Figure 33.** CH: 41, 44 and 52, the grid searching results of the two RBF parameters used in SVM.


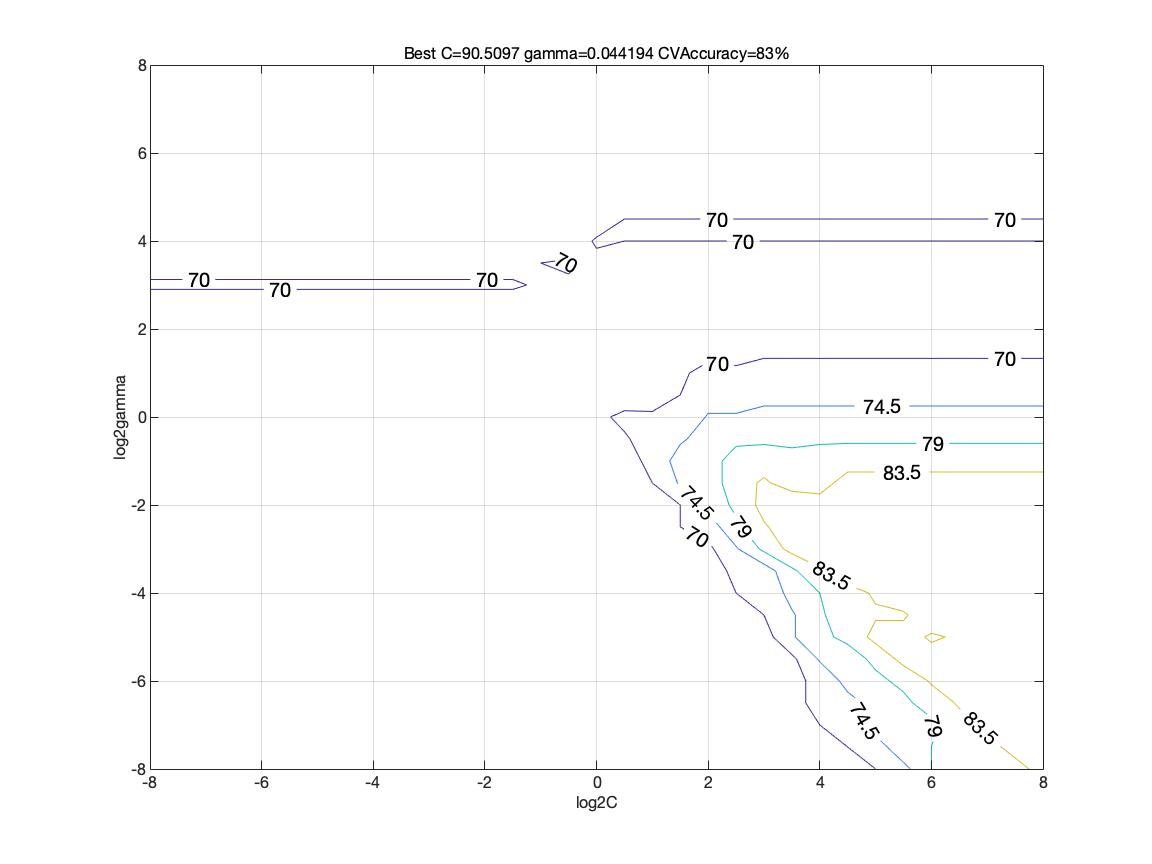


**Figure 34.** CH: 41, 50 and 52, the grid searching results of the two RBF parameters used in SVM.


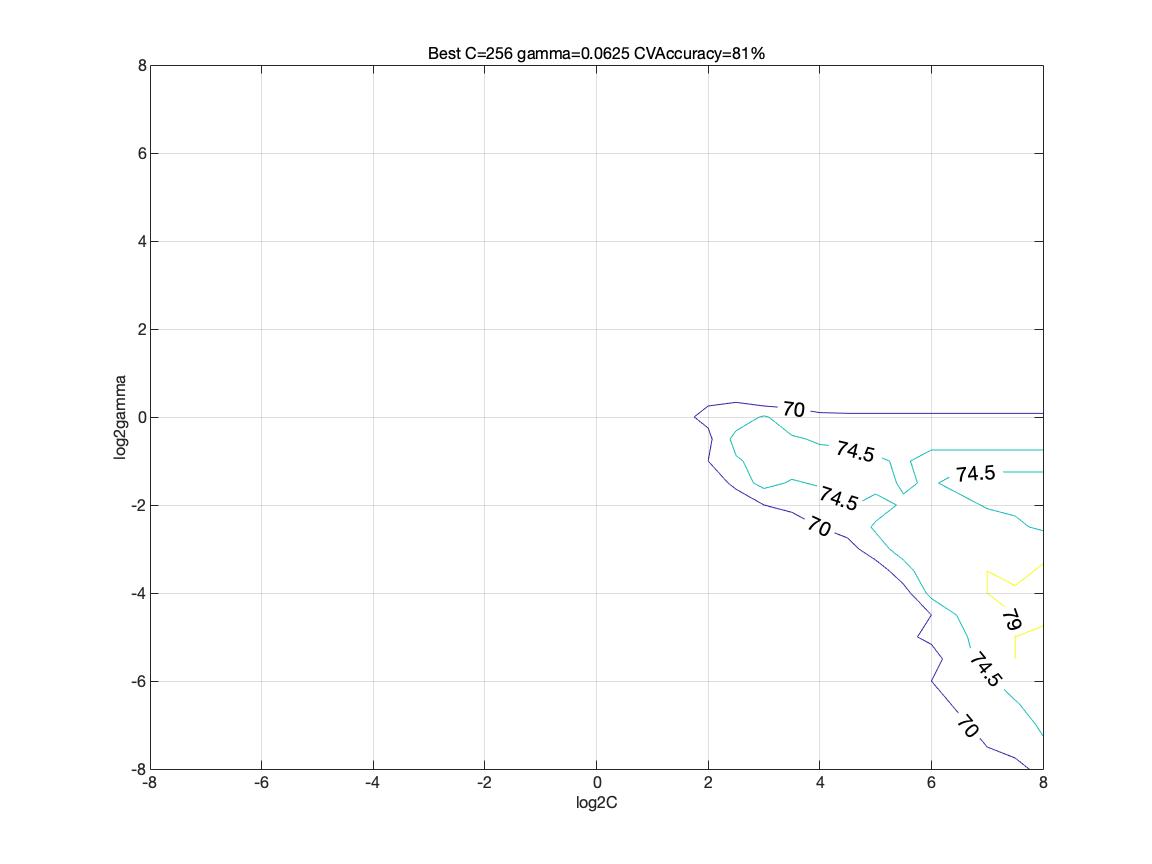


**Figure 35.** CH: 44, 50 and 52, the grid searching results of the two RBF parameters used in SVM.


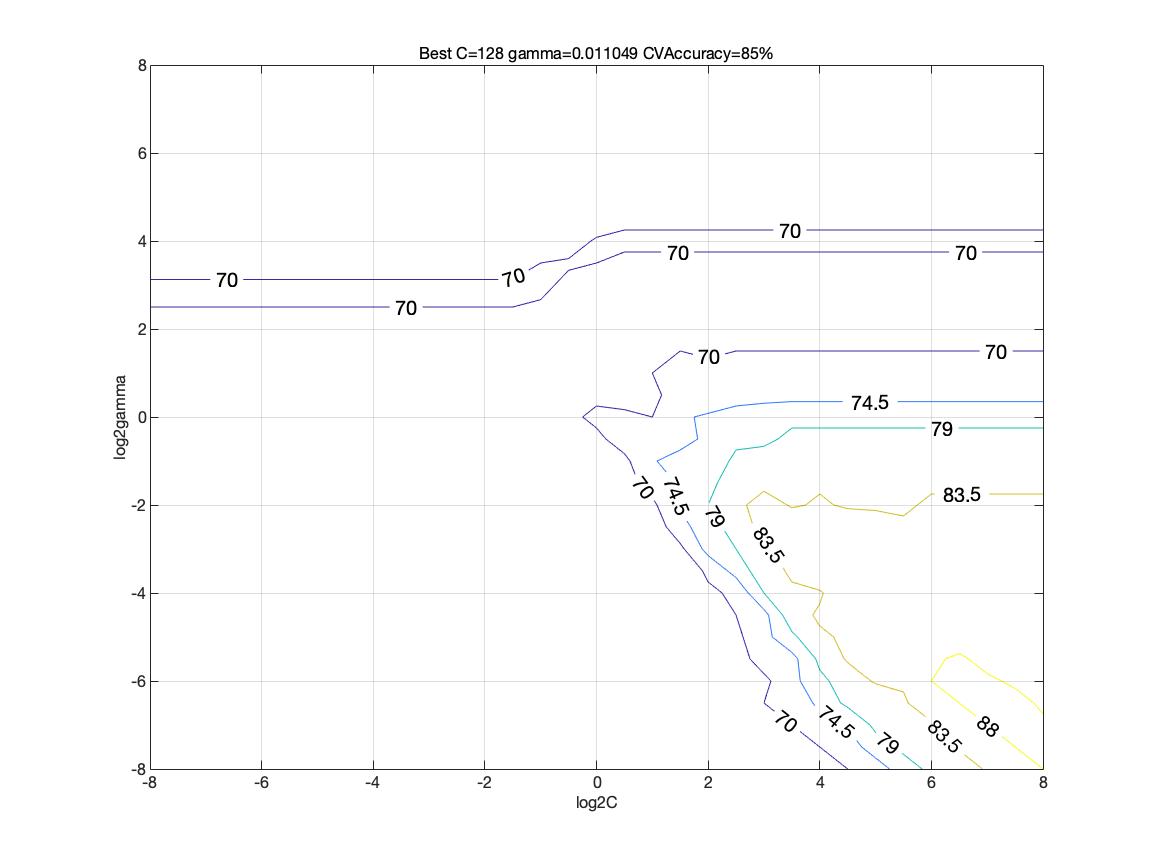


**Figure 36.** CH: 40, 41, 44 and 50, the grid searching results of the two RBF parameters used in SVM.


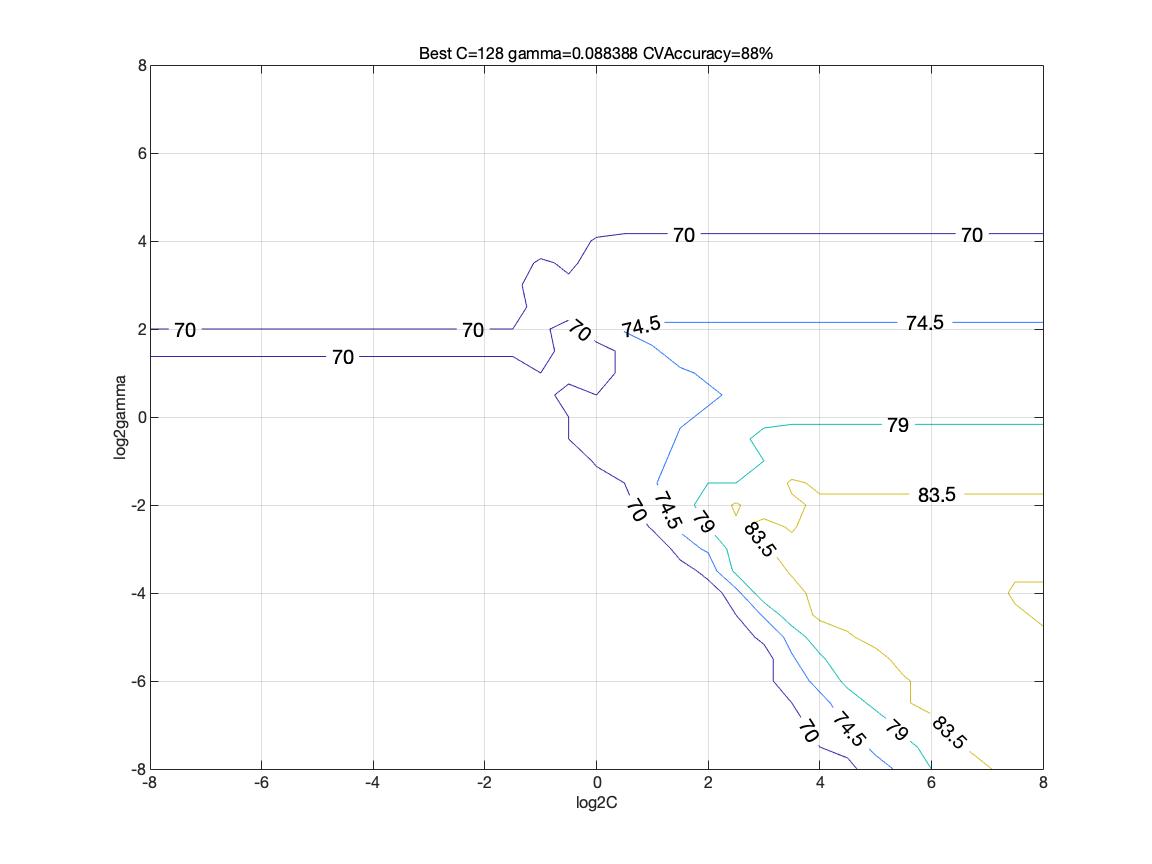


**Figure 37.** CH: 40, 41, 44 and 52, the grid searching results of the two RBF parameters used in SVM.


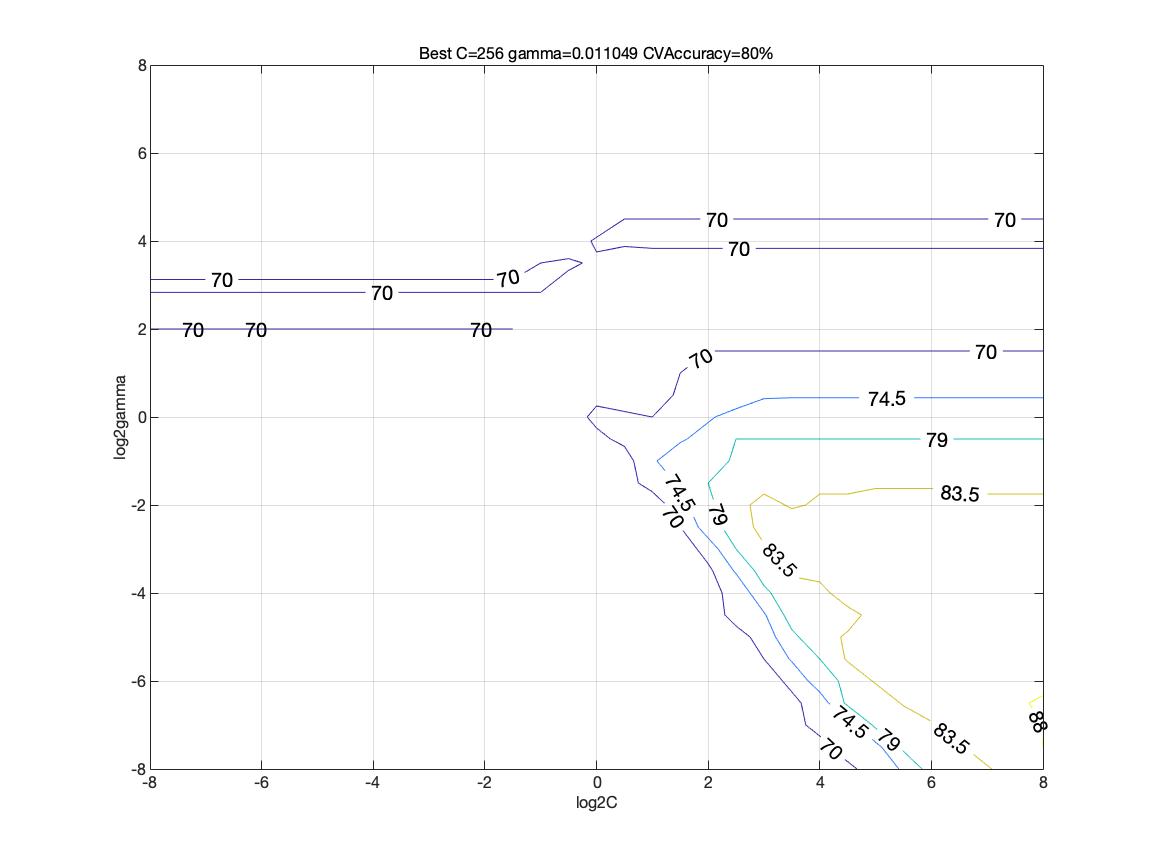


**Figure 38.** CH: 40, 41, 50 and 52, the grid searching results of the two RBF parameters used in SVM.


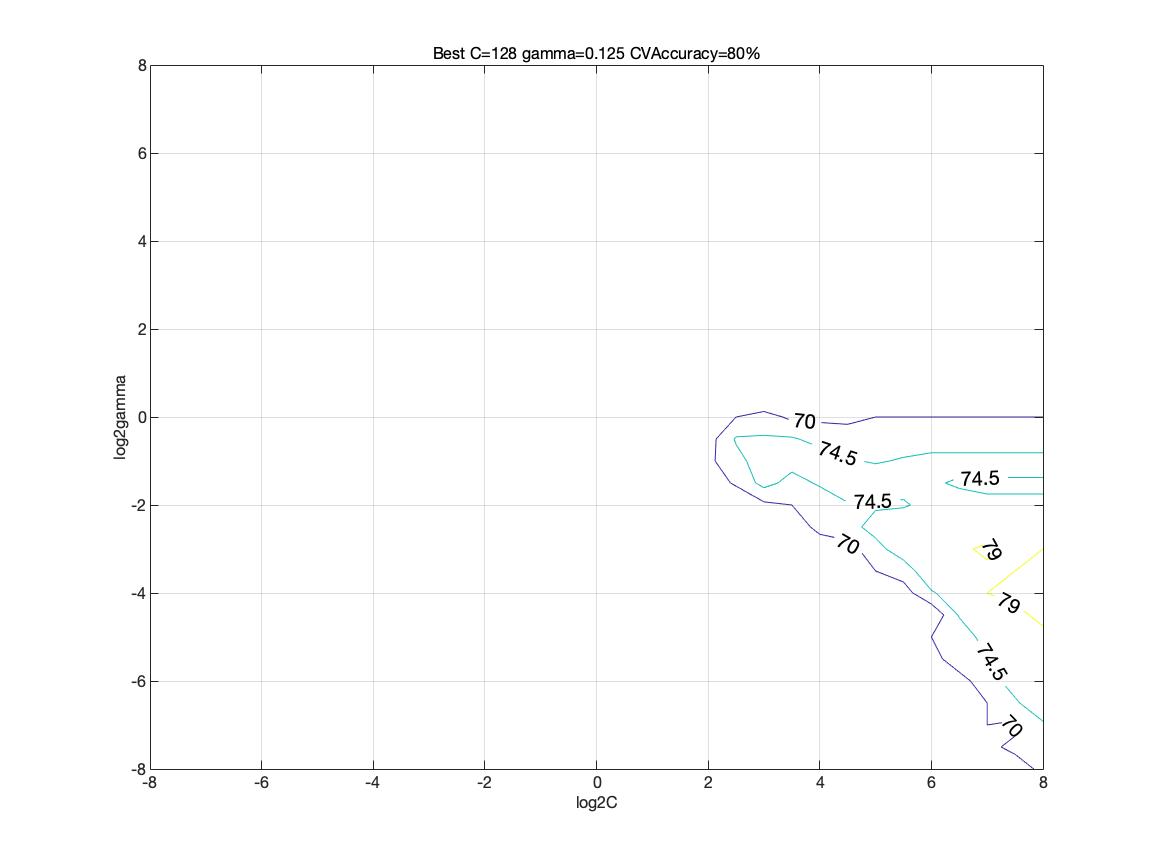


**Figure 39.** CH: 40, 44, 50 and 52, the grid searching results of the two RBF parameters used in SVM.


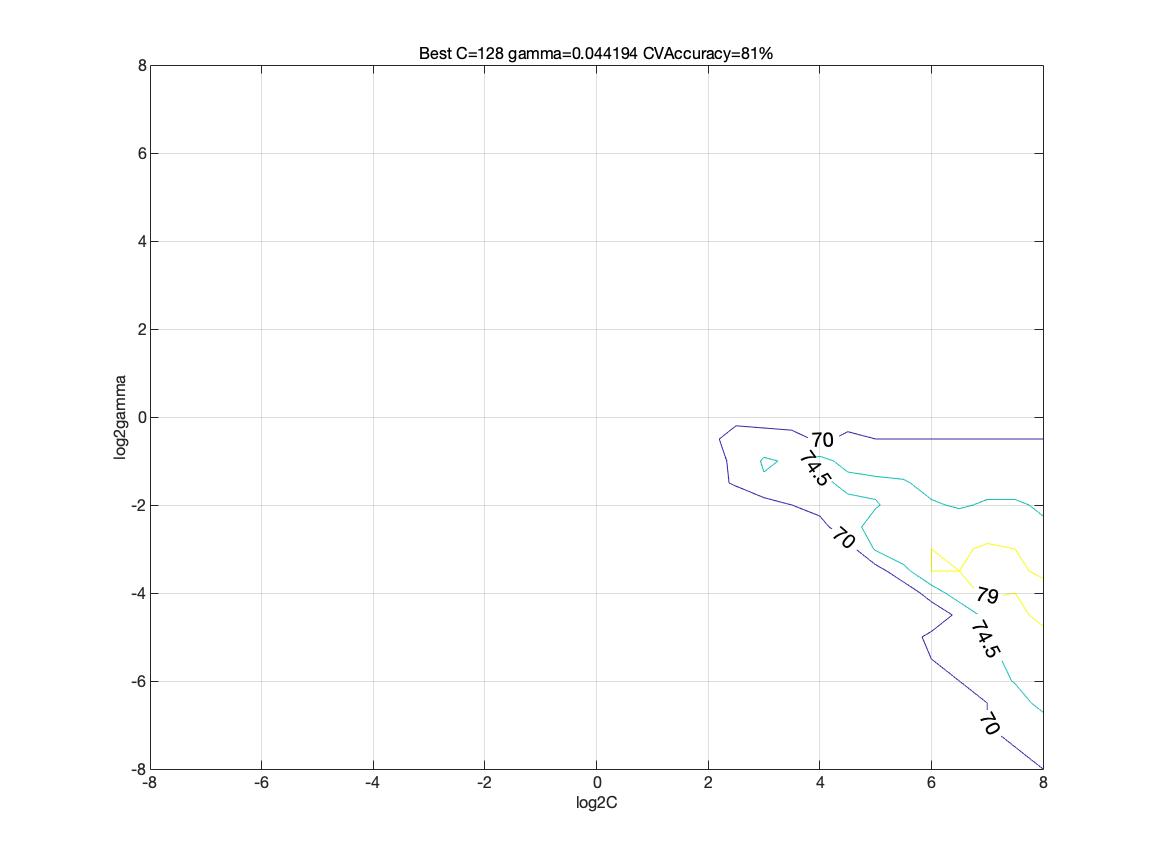


**Figure 40.** CH: 41, 44, 50 and 52, the grid searching results of the two RBF parameters used in SVM.


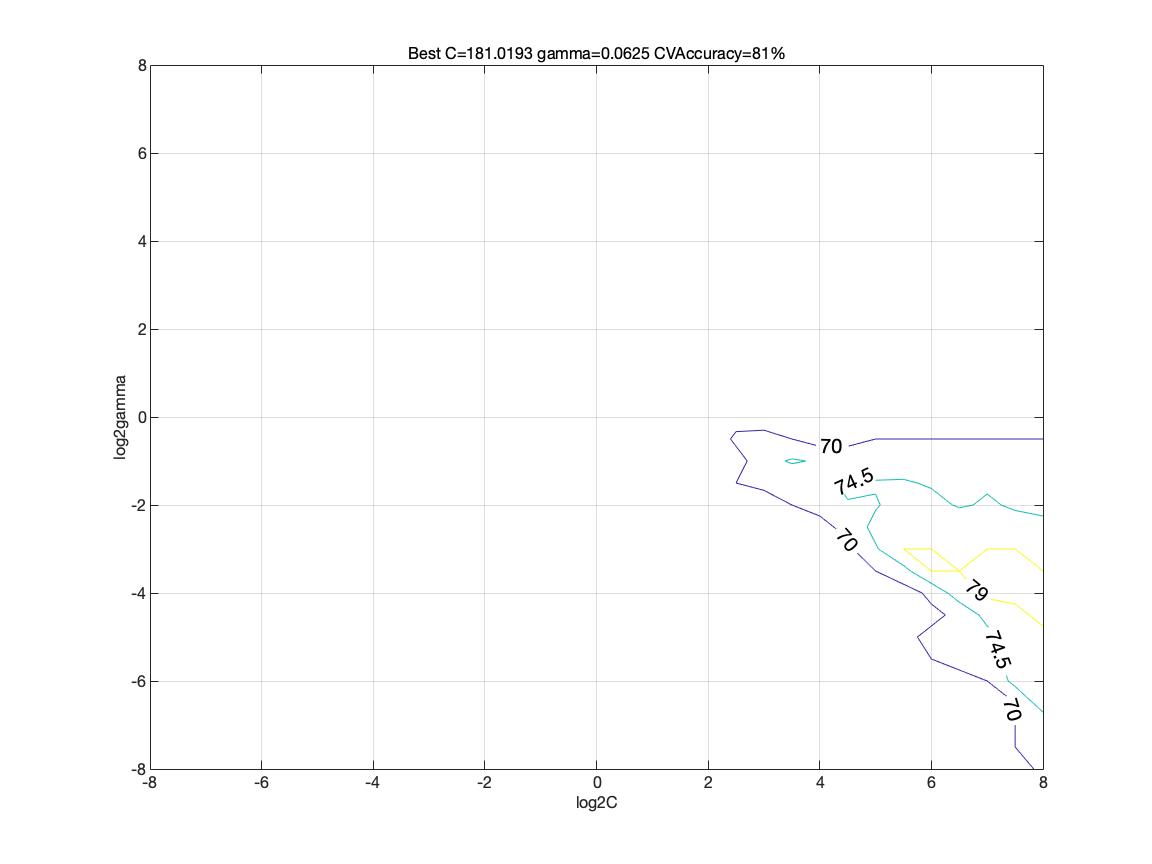


**Figure 41.** CH: 40, 41, 44, 50 and 52, the grid searching results of the two RBF parameters used in SVM.
